# High-resolution HLA phased haplotype frequencies to predict the success of unrelated donor searches and clinical outcome following hematopoietic stem cell transplantation

**DOI:** 10.1038/s41409-019-0520-6

**Published:** 2019-04-05

**Authors:** Stéphane Buhler, Helen Baldomero, Sylvie Ferrari-Lacraz, José Manuel Nunes, Alicia Sanchez-Mazas, Stravroula Massouridi-Levrat, Dominik Heim, Jörg Halter, Gayathri Nair, Yves Chalandon, Urs Schanz, Tayfun Güngör, Grazia Nicoloso, Jean-Marie Tiercy, Jakob Passweg, Jean Villard

**Affiliations:** 1 Transplantation Immunology Unit and National Reference Laboratory for Histocompatibility, Department of Diagnostic, Geneva University Hospitals, Geneva, Switzerland; 2 Anthropology Unit, AGP Laboratory, Department of Genetics and Evolution, University of Geneva, Geneva, Switzerland; 3 Division of Hematology, Basel University Hospital, Basel, Switzerland; 4 Institute of Genetics and Genomics in Geneva (iGE3), Geneva, Switzerland; 5 Division of Hematology, Department of Oncology, Geneva University Hospitals, Geneva, Switzerland; 6 Division of Hematology, University Hospital of Zurich, Zurich, Switzerland; 7 Department of Stem Cell Transplantation, University Children’s Hospital Zurich, Zurich, Switzerland; 8 Swiss Blood Stem Cells Registry, Swiss Transfusion SRC, Bern, Switzerland

**Keywords:** Bone marrow transplantation, Transplant immunology

## Abstract

HLA matching is a critical factor for successful allogeneic hematopoietic stem cell transplantation. For unrelated donor searches, matching is usually based on high-resolution typing at five HLA loci, looking for a 10/10 match. Some studies have proposed that further matching at the haplotype level could be beneficial for clinical outcome. In this study, we determined the phased haplotypes of 291 patients using family members and segregation analysis. The sum of ranks of the haplotypes carried by patients was used as a surrogate predictor of a successful unrelated donor search. The putative impact of haplotypes was then analyzed in a cohort of 211 recipients transplanted with 10/10 matched unrelated donors. A logistic regression analysis showed a highly significant effect of the haplotypes in the outcome of a search, but we did not find any significant effect on overall survival, graft versus host disease or relapse/progression following HSCT. This study provides useful data for the optimization of unrelated bone marrow donor searches, but does not confirm previous reports that matching at the haplotype level has a clinical impact following HSCT. Due to the extreme polymorphism of HLA genes, further studies are warranted to better understand the many factors at play.

## Introduction

Human leukocyte antigen (HLA) matching between recipients and donors is a prerequisite for successful allogeneic hematopoietic stem cell transplantation (HSCT), notably to avoid graft versus host disease (GVHD) as main post-transplant complication. Although new protocols for selecting donors are increasingly sought, even across the histocompatibility barrier [1–3], the gold standard is to look first for an HLA identical sibling. If such a genotypically identical sibling cannot be found, the preferred alternative is to search for a 10/10 or 12/12 phenotypically matched unrelated donor (MUD) [4, 5]. With unrelated donors, matching is based on high-resolution typing at HLA-A, B, C, DRB1, DQB1 and possibly DPB1 and DRB3/4/5 with no consideration given to putative haplotype matching between the recipient and his donor. However, even with well-matched unrelated donors, risks of transplant-related mortality are higher as compared to matched sibling donors because of minor histocompatibility antigens (mHA) spread across the whole genome and non-HLA linked polymorphisms (e.g. single nucleotide polymorphisms (SNPs), expression quantitative trait loci (eQTL), microsatellites) within the extended HLA region [6–12]. Thus, in an attempt to leverage such a hurdle, several studies have suggested a possible beneficial impact of HLA haplotype matching at reducing post-transplantation complications in patients transplanted with 10/10 MUD [13–16]. A haplotype defines which allele belongs to which copy of the two chromosomes, or alternatively, which alleles segregate together on a single chromosome. In practice, HLA haplotypes can either be phased unambiguously by family segregation analysis or imputed statistically from genotype data and HLA frequencies in populations. The underlying hypothesis is that if a recipient and his unrelated donor are matched for the same common haplotypes, such haplotypes could carry more conserved DNA segments shared by descent (including at nearby favorable SNPs) compared to rare haplotypes present in the population.

Besides the extremely high level of polymorphism, complex patterns of association define classical HLA genes. Some pairs like B~C and DRB1~DQB1 are found in tight association on chromosome 6 [17], whereas other loci are defined by weaker (HLA-A) or non-significant linkage (HLA–DPB1), due to more distant location or recombination hotspots [18, 19]. These characteristics represent a significant hindrance to determine HLA multi-locus haplotypes and their corresponding frequencies [20]. In consequence, powerful methodologies have been developed to assess haplotype frequencies in various populations [21, 22] and in large cohorts of unrelated donors [23], but such approaches need to rely on representative sample sizes and on assumptions that are not always met in practice [22]. In this context, the availability of families typed at several HLA loci for the purpose of HSCT-related donor searches has provided informative data to characterize HLA haplotypes by segregation analysis [24–26], contributing to define the probability of finding suitable unrelated donors [27–29] or to study clinical outcome of unrelated HSCT [13]. However, studies using phased haplotypes remain scarce in the literature and phased HLA haplotypes are needed in more populations because HLA frequencies vary significantly according to geography [30–32].

The first aim of this study was to investigate haplotype segregation in a large cohort of patients and their family living in Switzerland. This would allow us to constitute a reference panel to help in optimizing future unrelated donor searches for the significant proportion of patients in need of a transplantation with no HLA identical sibling [5]. The second aim was to use these phased haplotypes for predicting the outcome of unrelated donor searches for patients waiting for a HSCT. Within the last aim, we analyzed HSCT outcomes in a group of recipients transplanted with 10/10 MUD and we tested their HLA haplotypes frequencies as a potential relevant parameter in the clinical follow-up.

## Material and methods

### Patients

The phased haplotypes of individuals living in Switzerland were determined from the HLA-A, B and DRB1 typing of 843 patients and 2132 family members (Figure S1), allowing to constitute a cohort of 291 patients with high-resolution phased haplotypes. High-resolution typing was also performed at HLA-C and DQB1 for 290 of these patients as potential candidates for an unrelated donor search. Allogeneic HSCT was performed in 140 patients, including 101 with 10/10 MUD and 39 with mismatched donors. To enlarge the clinical cohort, 111 recipients of 10/10 MUD with family segregation data were obtained from the other Swiss transplant centers (Table S1). This study was approved by the ethical committee of the institution (CER 06-208 and 08-208R), and patients’ informed consents were obtained.

### Statistical analyses

#### HLA haplotypes

Haplotype frequencies were either estimated by direct counting on 291 patients based on segregating haplotypes or by using an implementation of the expectation–maximization (EM) algorithm on 6114 unrelated Swiss donors based on multi-locus unphased genotypes. Hardy–Weinberg (HW) equilibrium assumptions were assessed using a nested likelihood procedure. Global linkage disequilibrium between pairs of loci was tested using a resampling procedure and linkage disequilibrium for individual haplotypes was determined using standardized residuals. All these analyses were performed with the hla-net.eu Gene[RATE] tools [21, 33].

#### Unrelated donor searches

The sum of ranks of the phased haplotypes carried by each patient was considered as a surrogate predictor of a successful search (i.e. finding at least one 10/10 MUD). Haplotypes were ranked based on high-resolution haplotype frequencies estimated on 6114 donors from the Swiss registry (SBSC). The choice of the sum of haplotype ranks is analogous to a non-parametric approach with the goal of not relying directly and too heavily on estimated haplotype frequencies as search outcome determinants. Confusion matrices, logistic regression and receiver operating characteristic (ROC) curve were generated in R (version 3.5.0) using the packages ggplot2, reshape, caret and ROCR.

#### Clinical outcome

We followed the reasoning applied by Joris et al. [13] to consider that a low haplotype ranking in a recipient (i.e. carrying one or two frequent haplotypes) was a good proxy for haplotype matching with his 10/10 MUD. As unrelated donors are selected at a worldwide scale, SBSC frequencies were contrasted with frequencies estimated on donors from the United States [34]. Recipients were subdivided into categories based on the ranking of their haplotypes, using rank 50 and rank 20 as two distinct cut-offs to classify haplotypes as common or rare, and were subsequently analyzed in separate models for survival (see Tables S1 and S2, Table 2 and Fig. 2 for the categories considered). Obviously, the chosen cut-offs and categories are arbitrary to a degree, but this provided a compromise to the very heterogeneous values proposed in previous studies for defining common haplotypes [13, 15]. It also allowed to consider alternative groups while keeping sufficient and meaningful numbers of patients within each one. Furthermore, we would expect that a strong effect of haplotypes on clinical outcome should be robust and consistent across different cut-offs to be really considered as a relevant parameter. Secondary outcomes (relapse/progression, acute GVHD, chronic GVHD and survival status) were tested by univariate analyses and by estimating cumulative incidence. Cox proportional-hazards models were used to evaluate the effect of potential confounding variables, in addition to haplotypes, on overall survival, progression-free survival, relapse/progression and chronic GVHD. Because of missing dates for the onset of acute GVHD, a logistic regression was performed instead. The parameters considered were DPB1 matching, source of stem cells, year of treatment, type and stage of disease, patient age at transplantation, transplantation center, T-cell depletion, conditioning, cytomegalovirus (CMV) serological status and recipient/donor gender combination. Donor age was not analyzed because of missing data. HLA–DPB1 matching was also investigated as an explanatory variable for overall survival (OS), for occurrence of acute GVHD and for relapse/progression. These analyses were generated with SPSS on a total of 211 recipients (Table S1) with parameters equally distributed across groups except for recipient/donor gender (Table S2). The median waiting time was 116 days between donor search and transplantation with no significant difference among recipients according to haplotype groups.

## Results

### HLA haplotypes determination

A total of 420 distinct high-resolution HLA-A~B~DRB1 haplotypes were phased by segregation analysis in the 291 patients and HW equilibrium was not rejected at any locus. The most frequent haplotypes are listed in Table 1. None of them reached a frequency of 5% and only seven had a frequency >1%, with most haplotypes observed just twice or once in the cohort (Table S3). The three loci were not significantly associated to each other (*p*-value of 1 according to the likelihood-ratio test, no extreme value according to parametric resampling for global linkage disequilibrium). This was in agreement with the observation that only few haplotypes were in complete linkage across the three loci (Tables 1 and S3).

**Table 1:** Most frequent HLA-A~B~DRB1 phased haplotypes and linkage disequilibrium among allele pairs in the cohort of 291 patients

Haplotype	Freq.	Count	LD A-B	LD A-DRB1	LD B-DRB1	Rank SBSC	Rank NMDP EUR	Rank NMDP AFA	Rank NMDP API	Rank NMDP HIS
A*01:01~B*08:01~DRB1*03:01	0.0326	19	8.38	4.08	11.67	1	1	2	40	2
A*03:01~B*07:02~DRB1*15:01	0.0206	12	3.93	2.11	9.71	2	2	7	NA	3
A*02:01~B*07:02~DRB1*15:01	0.0172	10	0.31	0.73	9.71	5	4	41	615	9
A*03:01~B*35:01~DRB1*01:01	0.0137	8	4.31	2.96	8.38	4	8	149	75	23
A*29:02~B*44:03~DRB1*07:01	0.012	7	11.31	3.50	9.25	6	5	9	1257	1
A*01:01~B*57:01~DRB1*07:01	0.012	7	6.78	0.36	5.85	8	7	58	8	22
A*26:01~B*38:01~DRB1*13:01	0.0103	6	9.71	2.26	4.56	42	53	NA	NA	162
A*02:01~B*44:02~DRB1*04:01	0.0086	5	3.37	2.53	4.24	3	3	10	1292	46
A*30:01~B*13:02~DRB1*07:01	0.0086	5	11.98	3.98	7.17	10	10	178	4	16
A*24:02~B*07:02~DRB1*15:01	0.0086	5	1.14	0.67	9.71	13	13	337	183	72
A*02:01~B*51:01~DRB1*11:01	0.0086	5	2.38	0.08	3.47	15	28	122	496	27
A*01:01~B*08:01~DRB1*15:01	0.0086	5	8.38	−0.23	−0.17	25	25	544	NA	NA
A*24:02~B*08:01~DRB1*03:01	0.0086	5	0.34	0.63	11.67	47	34	1379	268	42
A*02:01~B*15:01~DRB1*04:01	0.0069	4	2.22	2.53	7.06	9	6	21	1419	39
A*02:01~B*18:01~DRB1*03:01	0.0069	4	0.22	0.17	2.82	68	71	80	NA	40
A*02:01~B*40:01~DRB1*13:02	0.0052	3	1.11	−0.35	3.16	7	9	81	NA	554
A*02:01~B*08:01~DRB1*03:01	0.0052	3	−1.86	0.17	11.67	17	11	20	NA	18
A*02:01~B*18:01~DRB1*11:04	0.0052	3	0.22	0.08	5.69	24	33	NA	NA	70
A*31:01~B*40:01~DRB1*04:04	0.0052	3	5.40	4.09	6.07	27	20	98	1264	137
A*02:01~B*51:01~DRB1*08:01	0.0052	3	2.38	0.55	1.78	29	73	245	NA	203
A*32:01~B*44:03~DRB1*07:01	0.0052	3	2.06	1.81	9.25	120	291	NA	236	234
A*01:01~B*15:17~DRB1*13:02	0.0052	3	4.04	2.70	8.41	210	327	NA	55	284
A*02:01~B*13:02~DRB1*13:01	0.0052	3	−0.04	0.09	1.15	333	NA	1131	NA	NA

LD: pairwise linkage disequilibrium as defined by standardized residuals; values ≥2 correspond to a significant association. Haplotypes in complete linkage (i.e. across the three loci) are shown in bold.

Rank of haplotypes estimated in 6114 volunteer donors from the Swiss registry (SBSC) and in four large groups (EUR: donors of European descent, AFA: donors of African descent, API: donors of Asian descent, HIS: donors of South American descent) of volunteer donors from the National Marrow Donor Program (NMDP) (ref. ^34^)

*NA* not available

As family data are seldom available to confirm haplotype frequencies when samples are typed for HLA, we also estimated haplotype frequencies on our data by using the EM algorithm without accounting for phase information and we compared the results with those of our segregation analysis. It showed us that 265 haplotypes were simultaneously assigned by both approaches, while 155 were assigned by segregation analysis only and 258 were found by EM only. This represented a low concordance (39%) between the two approaches. In addition, the haplotypes assigned by both approaches sometimes exhibited frequency differences. As an example, haplotype A*02:01~B*08:01~DRB1*03:01 exhibits a frequency of 0.52% through segregation analysis, whereas it reaches an overestimated frequency of 1.53% through EM; this is because its alleles frequently occur simultaneously at the genotype level but are most often not linked on the same chromosome (Figure S2).

The HLA-A~B~DRB1 phased haplotypes in the 291 patients were ranked according to their frequencies estimated on the 6114 Swiss volunteer donors (Tables 1 and S3). Both sets of data were cross-tabulated in order to predict the most probable extended HLA haplotypes in Switzerland as listed in Table S4.

### Predicting 10/10 matched unrelated donors searches

**Fig. 1:**
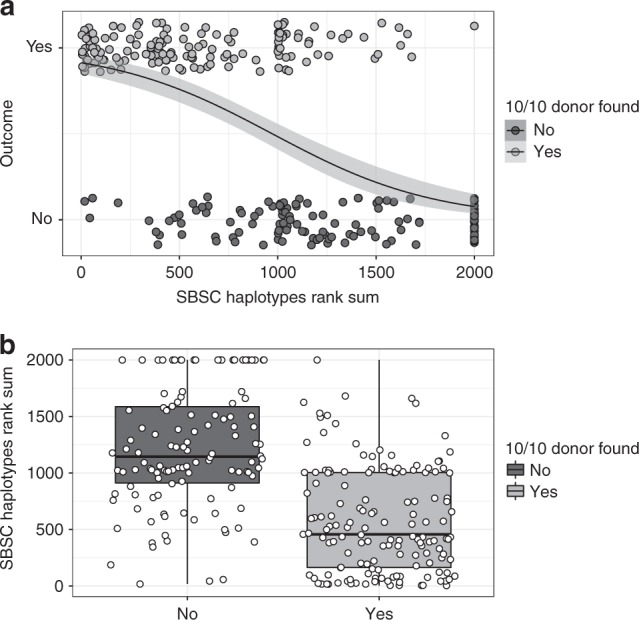
Unrelated search outcome with the sum of haplotype ranks used as an explanatory variable, **a** logistic regression on the data represented by the black line with confidence interval in light gray, **b** box-and-whisker plots

Using a logistic regression model with the sum of ranks as an explanatory variable (Fig. 1a), we could show a significant association to search outcome (*p* = 2.25e−14). We then investigated the best rank cut-off to predict search outcome with good sensibility and specificity, and the inspection of boxplots (Fig. 1b) suggested a sum of 1000, i.e. corresponding to patients carrying at least one very rare haplotype not seen in SBSC or carrying two infrequent haplotypes. To confirm this preliminary assessment, we ran an ROC curve analysis, which showed that a sum of 1000 was a good trade-off between true and false-positive rates (Figure S3). Using this cut-off value of 1000, we achieved a sensitivity of 0.71 and a specificity of 0.72. Most false positives (i.e. no 10/10 MUD found despite a sum <1000) were due to patients carrying rare allele(s) or unusual B~C or DRB1~DQB1 association(s), which significantly impaired the chances of finding a donor. By contrast, false negatives (i.e. at least one 10/10 MUD found despite a sum >1000) were often observed in patients carrying one very frequent haplotype besides the rare one, explaining why a donor could still be found in this specific constellation.

### HLA haplotypes and clinical outcome following HSCT

**Fig. 2:**
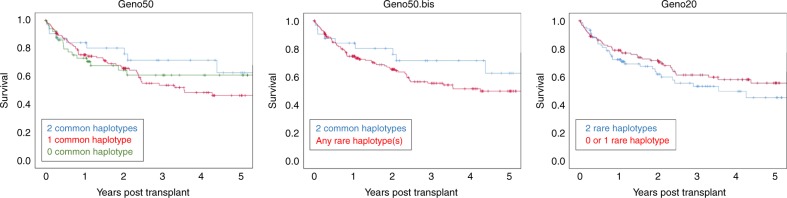
Kaplan–Meier plots for the different genotype categories considered regarding haplotype frequency and HSCT outcome. Geno50: recipients carrying 2, 1 or 0 common haplotypes with a frequency ≤ rank 50; geno50.bis: recipients carrying 2 common haplotypes with a frequency ≤ rank 50 versus recipients carrying any rare haplotypes with a frequency > rank 50; geno20: recipients carrying 0 or 1 rare haplotype versus recipients carrying 2 rare haplotypes with a frequency > rank 20

The univariate analyses did not reveal a significant effect of haplotypes on overall survival (Table 2 and Fig. 2) or on other outcomes such as GVHD (Table 2). Moreover, cumulative incidence for relapse and GVHD was not different across haplotype groups (Figure S4). By contrast, better DPB1 matching was slightly, although significantly, associated to less acute GVHD (*p* = 0.02) and higher relapse/progression rate (*p* = 0.03), but not to better overall survival (Table 2).

**Table 2:** Clinical outcome following HSCT: summary of univariate analyses

Outcome	Explanatory variable	Test	Statistic	df	*p*-Value
Overall survival (OS)	geno50	Log-Rank	1.72	2	0.42
	geno50.bis	Log-Rank	1.54	1	0.22
	geno20	Log-Rank	1.21	1	0.27
Relapse/progression	geno50	*χ*^2^	2.32	2	0.31
	geno50.bis	Fisher	–	–	0.68 (two-sided)
	geno20	Fisher	–	–	>0.99 (two-sided)
aGVHD	geno50	*χ*^2^	0.69	4	0.95
	geno50.bis	*χ*^2^	0.5	2	0.78
	geno20	*χ*^2^	0.63	2	0.73
cGVHD	geno50	*χ*^2^	1.38	2	0.5
	geno50.bis	Fisher	–	–	0.83 (two-sided)
	geno20	Fisher	–	–	0.27 (two-sided)
Survival status at this date	geno50	*χ*^2^	7.55	4	0.11
	geno50.bis	*χ*^2^	6.59	2	0.04
	geno20	*χ*^2^	1.08	2	0.58
Overall survival (OS)	DPB1 matching	Log-Rank	0.52	2	0.77
aGVHD	DPB1 matching	*χ*^2^	7.64	2	0.02
Relapse/progression	DPB1 matching	*χ*^2^	7.01	2	0.03

Geno50: recipients carrying 2, 1 or 0 common haplotypes with a frequency ≤ rank 50; geno50.bis: recipients carrying 2 common haplotypes with a frequency ≤ rank 50 versus recipients carrying any rare haplotypes with a frequency > rank 50; geno20: recipients carrying 0 or 1 rare haplotype versus recipients carrying 2 rare haplotypes with a frequency > rank 20

*aGVHD* acute graft vesus host disease, *cGVHD* chronic graft versus host disease, *df* degrees of freedom

Haplotypes were never significant in multivariate analyses. Recipients’ age, stage of disease and transplantation center were significantly associated to survival (Tables 3 and S5), progression-free survival and relapse/progression (results not shown). In models inspected, older recipients and/or recipients with an advanced disease had a lower chance of survival and progression-free survival but a higher risk of relapse/progression. Furthermore, in agreement with the results obtained in the univariate setting, DPB1 matching was not associated to better survival (Tables 3 and S5), but was a significant risk factor for relapse/progression and progression-free survival when not accounting for conditioning (results not shown). The other variables considered were never found to be significant, except type of disease in the models for relapse/progression because of a higher risk in recipients suffering from acute leukemia. Regarding chronic GVHD, no variable was significant. Logistic regression for acute GVHD revealed small but significant effects of T-cell depletion and source of cells and minor differences between acute leukemia and the other diseases (results not shown).

**Table 3:** Cox regression model for overall survival with geno50

							95.0% CI for Exp(B)	
Explanatory variable	*B*	SE	Wald	df	Sig.	Exp(B)	Lower	Upper
TX center			22.715	3	0			
2	−0.592	0.344	2.965	1	0.085	0.553	0.282	1.085
3	1.017	0.31	10.754	1	0.001	2.765	1.506	5.078
4	0.789	0.69	1.309	1	0.253	2.201	0.57	8.509
Age			10.77	3	0.013			
Age (20–40)	1.02	0.543	3.528	1	0.06	2.773	0.957	8.041
Age (40–60)	1.03	0.473	4.735	1	0.03	2.801	1.108	7.081
Age (>60)	1.688	0.528	10.206	1	0.001	5.409	1.92	15.236
Disease stage			11.286	2	0.004			
Disease stage (intermediate)	0.268	0.285	0.888	1	0.346	1.308	0.748	2.285
Disease stage (advanced)	1.042	0.317	10.828	1	0.001	2.835	1.524	5.272
DPB1 MM			0.628	2	0.731			
DPB1 MM (1 MM)	0.084	0.319	0.07	1	0.791	1.088	0.583	2.032
DPB1 MM (2 MM)	−0.145	0.353	0.169	1	0.681	0.865	0.433	1.729
geno50			0.805	2	0.669			
geno50 (1 common haplotype)	0.291	0.377	0.595	1	0.44	1.337	0.639	2.799
geno50 (0 common haplotype)	0.376	0.428	0.771	1	0.38	1.456	0.63	3.368

Baseline for TX center = 1, for age = <20, for disease stage = early, for DPB1 MM = 0 MM, for geno50 = 2 common haplotypes. *MM* mismatch, *TX* transplant

## Discussion

HLA haplotype determination is usually not based on family segregation, but relies on estimations performed with state-of-the-art EM algorithm implementations [35, 36]. Interestingly, the comparative analysis undertaken in this study showed that only a mere 39% assignation concordance was achieved between the real phased haplotypes in our cohort and a “blind” estimation with the EM algorithm. The discrepancies were mostly due to rare haplotypes and this problem has recently been discussed [20], but this also concerned the frequent haplotype A*02:01~B*08:01~DRB1*03:01. This illustrates the usefulness of family data for characterizing high-resolution multi-locus haplotypes [26] when sample sizes are not huge (meaning hundred thousand, or even millions of individuals). In addition, the use of next-generation sequencing (NGS) technologies is expected to increase the variability of high-resolution haplotypes. For instance, our data at third-field-level resolution includes A*02:01:01~B*08:01:01~DRB1*03:01:01 and A*02:01:01~B*08:01:02~DRB1*03:01:01 haplotypes.

In the unrelated setting, the probability of finding a 10/10 matched donor is largely determined by haplotype frequencies, our analyses thus agree with previous publications [13, 27–29, 37–39]. Accurate prediction allows to define the optimal strategy to find the best suitable donor, whether a matched unrelated, mismatched unrelated or a haploidentical donor [40, 41].

By exploring several different models, we show that the presence of frequent haplotype(s) in the patients had no impact on HSCT outcome and thus partly differ from previous studies. Notably, Petersdorf et al. [15] found that haplotype matching between donors and recipients was associated with less grade 3–4 acute GVHD and with a higher risk of disease recurrence, but not with overall survival. In their publication, however, the haplotypes were determined in both the recipients and their MUDs with a DNA microarray method that defines the physical linkage between HLA-A, B and DRB1 alleles, whereas our study determined them from family segregation analysis in recipients with indirect imputation of haplotype matching with their MUDs. A second difference between the two studies is the over representation in the former of the frequent haplotype A*01:01~B*08:01~DRB1*03:01, which is found in about 11% of the 246 donor–recipient pairs analyzed and which is always matched except in three cases, thus strongly contributing statistically to the results. In our cohort, this haplotype is present in only 6% of the 211 recipients. A second publication based on a large Japanese cohort [14] showed that among the three major conserved extended haplotypes (HP-P1, 2 and 3) found in Japanese, HP-P2 significantly reduced the risk of grade 2–4 acute GVHD, while HP-P3 tended to increase this risk, suggesting that conserved haplotypes may be beneficial or deleterious for the clinical outcome. In a third publication [13], an effect of haplotypes was seen only on the incidence of ≥grade 2 acute GVHD (survival or relapse were not significant) and only in the category “1 or 2 frequent haplotype(s) (FH)” (but not in the categories 1 FH and 2 FH taken individually). Moreover, the “1 of 2 FH” category was not associated with GVHD in univariate analysis and was only significant (*p* = 0.026) when adjusting for other factors in a multivariate model.

The controversial effect of haplotype matching might be related to methodological heterogeneity at defining common haplotypes, limitations to achieve sufficient statistical power and perhaps more likely to different impacts of individual haplotypes.

In 10/10 matched unrelated transplantation, the role of HLA and non-HLA genes is critical in the pathophysiology of GVHD and other outcomes [7, 10, 42]. HLA–DPA1 and DPB1, which are usually not in linkage disequilibrium with the other HLA genes [18, 19], are often not considered to be part of haplotypes and are not systematically characterized during donor selection [5], although the role of DPB1 has been well documented [43–45]. In our study, DPB1 mismatching is significantly associated with acute GHVD as an independent factor, albeit only in the univariate setting (Table 2). The lack of linkage between DPB1 and other loci observed even in high-frequency haplotypes might account for the difficulty in demonstrating an impact of these haplotypes on clinical outcome.

Furthermore, the presence of polymorphisms in the so called “identical HLA haplotypes” is demonstrated by routine typing with NGS, because this methodology reveals many new polymorphisms in exons not encoding the peptide binding region, as well as in non-coding regions. There is no reason to believe that the situation is different outside HLA genes across chromosome 6. Indeed, the impact of non-HLA genes (e.g. cytokines, cytokine receptors) and polymorphisms such as microsatellites and SNPs has been reported to influence clinical outcome in unrelated HSCT [6, 8, 9, 11, 12, 46]. Although SNPs can directly affect the sequences of immunogenic peptides leading to mHA disparities, they may also modify genes encoding proteins involved in the pathophysiology of GVHD, such as TNF alpha, complement, TAP1/2, LMP1/7.

One important limitation of our study resides in the small number of transplanted patients, although it is close to the number of patients included in the seminal study of Petersdorf et al., which did not allow testing the putative effect(s) of individual haplotypes. Moreover, because the Swiss population is highly heterogeneous, we may lack statistical power to detect modest effects of haplotype matching. Therefore, our results can perhaps not be generalized to countries characterized by lower levels of diversity.

In conclusion, our study establishes the list of haplotypes observed in Switzerland, which shows a high population diversity despite its small size [30]. As expected, we observe that frequent haplotypes are strongly associated with a high probability to find a 10/10 matched unrelated donor. On the other hand, our results support the hypothesis that haplotype matching does not impact the clinical outcome and that more evidences are needed to better understand the numerous factors involved.

## Supplementary information

Table S1

Table S2

Table S3

Table S4

Table S5

Supplementary Figures legends

Figure S1

Figure S2

Figure S3

Figure S4
