# Correction: High-resolution HLA phased haplotype frequencies to predict the success of unrelated donor searches and clinical outcome following hematopoietic stem cell transplantation

**DOI:** 10.1038/s41409-020-0862-0

**Published:** 2020-05-04

**Authors:** Stéphane Buhler, Helen Baldomero, Sylvie Ferrari-Lacraz, José Manuel Nunes, Alicia Sanchez-Mazas, Stravroula Massouridi-Levrat, Dominik Heim, Jörg Halter, Gayathri Nair, Yves Chalandon, Urs Schanz, Tayfun Güngör, Grazia Nicoloso, Jean-Marie Tiercy, Jakob Passweg, Jean Villard

**Affiliations:** 1 Transplantation Immunology Unit and National Reference Laboratory for Histocompatibility, Department of Diagnostic, Geneva University Hospitals, Geneva, Switzerland; 2 Anthropology Unit, AGP Laboratory, Department of Genetics and Evolution, University of Geneva, Geneva, Switzerland; 3 Division of Hematology, Basel University Hospital, Basel, Switzerland; 4 Institute of Genetics and Genomics in Geneva (iGE3), Geneva, Switzerland; 5 Division of Hematology, Department of Oncology, Geneva University Hospitals, Geneva, Switzerland; 6 Division of Hematology, University Hospital of Zurich, Zurich, Switzerland; 7 Department of Stem Cell Transplantation, University Children’s Hospital Zurich, Zurich, Switzerland; 8 Swiss Blood Stem Cells Registry, Swiss Transfusion SRC, Bern, Switzerland

**Keywords:** Bone marrow transplantation, Transplant immunology

**Correction to:** **Bone Marrow Transplantation** (2019) 54: 1701–1709

10.1038/s41409-019-0520-6

published online 05 April 2019

This Article was originally published under Nature Research’s License to Publish, but has now been made available under a [CC BY 4.0] license. The PDF and HTML versions of the Article have been modified accordingly.

