# Supplementary material for: High-resolution HLA phased haplotype frequencies to predict the success of unrelated donor searches and clinical outcome following hematopoietic stem cell transplantation
Source: Bone Marrow Transplant. 2019 Apr 5;54(10):1701–9. doi: 10.1038/s41409-019-0520-6 (PMC7198472; doi:10.1038/s41409-019-0520-6)
Supplement: Supplementary file 1 — Table S1 [file 41409_2019_520_MOESM1_ESM.docx]

| **Recipient's characteristics** | **N** |  | **Graft parameters** | **N** |  | **HSCT outcomes** | **N** |
| --- | --- | --- | --- | --- | --- | --- | --- |
| *Disease:* |  |  | *TX center:* |  |  | *Relapse/progression:* |  |
| Acute leukemia | 100 |  | 1 | 65 |  | no | 152 |
| Myelodysplastic/myeloproliferative syndromes | 42 |  | 2 | 52 |  | yes | 59 |
| Lymphoid malignancy | 25 |  | 3 | 61 |  |  |  |
| Nonmalignant disorders | 24 |  | 4 | 33 |  | *aGVHD grade:* |  |
| Plasma cell disorders | 14 |  |  |  |  | 0 | 90 |
| Chronic myeloid leukemia | 6 |  | *Year of treatment:* |  |  | 1 | 45 |
|  |  |  | 2008 | 7 |  | 2-4 | 55 |
| *Disease stage:* |  |  | 2009 | 20 |  | NA | 21 |
| early | 99 |  | 2010 | 15 |  |  |  |
| intermediate | 72 |  | 2011 | 30 |  | *cGVHD:* |  |
| advanced | 40 |  | 2012 | 26 |  | no | 155 |
|  |  |  | 2013 | 32 |  | yes | 56 |
| *Age:* |  |  | 2014 | 35 |  |  |  |
| <20 yo | 51 |  | 2015 | 37 |  | *Survival status at this date:* |  |
| 20-40 yo | 38 |  | 2016 | 9 |  | Dead | 77 |
| 40-60 yo | 84 |  |  |  |  | Alive | 133 |
| >60 yo | 38 |  | *Source of cells:* |  |  | Died before HSCT but after conditioning | 1 |
|  |  |  | bone marrow | 46 |  |  |  |
| *HLA haplotypes:* |  |  | peripheral blood stem cells | 165 |  |  |  |
| *geno50 (based on rank 50)* |  |  |  |  |  |  |  |
| 2 common haplotypes | 34 |  | *DPB1 matching between recipient and donor:* |  |  |  |  |
| 1 common haplotype | 126 |  | 0 MM | 44 |  |  |  |
| 0 common haplotype | 52 |  | 1 bidirectional MM | 64 |  |  |  |
| *geno50.bis (based on rank 50)* |  |  | 1 graft versus host MM | 22 |  |  |  |
| 2 common haplotypes | 34 |  | 1 rejection MM | 20 |  |  |  |
| any rare haplotype(s) | 178 |  | 2 MM | 59 |  |  |  |
| *geno20 (based on rank 20)* |  |  | NA | 3 |  |  |  |
| 0 or 1 rare haplotype | 122 |  |  |  |  |  |  |
| 2 rare haplotypes | 90 |  |  |  |  |  |  |
| aGVHD: acute graft versus host disease; cGVHD: chronic graft versus host disease; MM: mismatch(s); NA: not available; N : sample size | | | | | | | |

**Table S1** Main characteristics of the clinical cohort (n=211 transplanted patients)
