# Supplementary material for: High-resolution HLA phased haplotype frequencies to predict the success of unrelated donor searches and clinical outcome following hematopoietic stem cell transplantation
Source: Bone Marrow Transplant. 2019 Apr 5;54(10):1701–9. doi: 10.1038/s41409-019-0520-6 (PMC7198472; doi:10.1038/s41409-019-0520-6)
Supplement: Supplementary file 2 — Table S2 [file 41409_2019_520_MOESM2_ESM.docx]

|  | **geno50** | | |  | **geno20** | |  |
| --- | --- | --- | --- | --- | --- | --- | --- |
|  | 2 common | 1 common | 0 common | Chi square p value | 0 or 1 rare | 2 rare | Chi square p value |
| **Disease stage** |  |  |  |  |  |  |  |
| early | 19 | 60 | 20 | 0.505 | 64 | 35 | 0.113 |
| intermediate | 8 | 43 | 21 |  | 38 | 34 |  |
| late | 6 | 23 | 11 |  | 19 | 21 |  |
| **Patients age at HSCT** |  |  |  |  |  |  |  |
| <20 | 8 | 33 | 10 | 0.229 | 28 | 23 | 0.713 |
| 20-40 | 6 | 20 | 12 |  | 21 | 17 |  |
| 40-60 | 16 | 44 | 24 |  | 47 | 37 |  |
| >60 | 3 | 29 | 6 |  | 25 | 13 |  |
| **Stem cell source** |  |  |  |  |  |  |  |
| BM | 4 | 31 | 11 | 0.3 | 23 | 23 | 0.255 |
| PBSC | 29 | 95 | 41 |  | 98 | 67 |  |
| **Conditioning** |  |  |  |  |  |  |  |
| RIC | 15 | 63 | 20 | 0.371 | 57 | 41 | 0.823 |
| MAC | 18 | 63 | 32 |  | 64 | 49 |  |
| **T cell depletion** |  |  |  |  |  |  |  |
| No | 28 | 116 | 49 | 0.298 | 108 | 85 | 0.182 |
| Yes | 5 | 10 | 3 |  | 13 | 5 |  |
| **Recipient/donor gender** |  |  |  |  |  |  |  |
| M/M | 15 (-0.18) | 60 (0.25) | 24 (-0.13) | 0.037 | 62 (1.46) | 37 (-1.46) | 0.01 |
| F/M | 3 (-1.75) | 23 (-0.93) | 17 (2.54) |  | 15 (-3.34) | 28 (3.34) |  |
| M/F | 11 (2.49) | 22 (-0.25) | 5 (-1.81) |  | 25 (1.16) | 13 (-1.16) |  |
| F/F | 4 (-0.45) | 21 (0.99) | 6 (-0.74) |  | 19 (0.48) | 12 (-0.48) |  |
| **CMV status** |  |  |  |  |  |  |  |
| D-/R- | 11 | 50 | 17 | 0.712 | 53 | 25 | 0.05 |
| D+/R- | 6 | 14 | 11 |  | 16 | 15 |  |
| D+/R+ | 9 | 34 | 15 |  | 26 | 32 |  |
| D-/R+ | 7 | 26 | 9 |  | 25 | 17 |  |
| **DPB1 matching** |  |  |  |  |  |  |  |
| 0 MM | 12 | 25 | 7 | 0.15 | 32 | 12 | 0.106 |
| 1 MM | 18 | 61 | 27 |  | 59 | 47 |  |
| 2 MM | 4 | 38 | 17 |  | 29 | 30 |  |
| not determined | 0 | 2 | 1 |  | 2 | 1 |  |
| BM: bone marrow, PBSC: peripheral blood stem cells, RIC: reduced intensity conditioning, MAC: myeloablative conditioning, M: male, F: female, D: donor, R: recipient, MM: mismatch. | | | | | | | |

**Table S2** Distribution of the main variables considered in multivariate analyses (MVA) in the cohort of 211 transplanted patients and across haplotype groups (standardized residuals are shown within parentheses for variables with a p value < 0.05)
