# Supplementary material for: High-resolution HLA phased haplotype frequencies to predict the success of unrelated donor searches and clinical outcome following hematopoietic stem cell transplantation
Source: Bone Marrow Transplant. 2019 Apr 5;54(10):1701–9. doi: 10.1038/s41409-019-0520-6 (PMC7198472; doi:10.1038/s41409-019-0520-6)
Supplement: Supplementary file 3 — Table S3 [file 41409_2019_520_MOESM3_ESM.docx]

| Haplotype | freq | count | LD A-B | LD A-DRB1 | LD B-DRB1 | Rank SBSC | Rank NMDP EUR | Rank NMDP AFA | Rank NMDP API | Rank NMDP HIS |
| --- | --- | --- | --- | --- | --- | --- | --- | --- | --- | --- |
| **A*01:01~B*08:01~DRB1*03:01** | 0.0326 | 19 | 8.38 | 4.08 | 11.67 | 1 | 1 | 2 | 40 | 2 |
| **A*03:01~B*07:02~DRB1*15:01** | 0.0206 | 12 | 3.93 | 2.11 | 9.71 | 2 | 2 | 7 | NA | 3 |
| A*02:01~B*07:02~DRB1*15:01 | 0.0172 | 10 | 0.31 | 0.73 | 9.71 | 5 | 4 | 41 | 615 | 9 |
| **A*03:01~B*35:01~DRB1*01:01** | 0.0137 | 8 | 4.31 | 2.96 | 8.38 | 4 | 8 | 149 | 75 | 23 |
| **A*29:02~B*44:03~DRB1*07:01** | 0.012 | 7 | 11.31 | 3.50 | 9.25 | 6 | 5 | 9 | 1257 | 1 |
| A*01:01~B*57:01~DRB1*07:01 | 0.012 | 7 | 6.78 | 0.36 | 5.85 | 8 | 7 | 58 | 8 | 22 |
| **A*26:01~B*38:01~DRB1*13:01** | 0.0103 | 6 | 9.71 | 2.26 | 4.56 | 42 | 53 | NA | NA | 162 |
| **A*02:01~B*44:02~DRB1*04:01** | 0.0086 | 5 | 3.37 | 2.53 | 4.24 | 3 | 3 | 10 | 1292 | 46 |
| **A*30:01~B*13:02~DRB1*07:01** | 0.0086 | 5 | 11.98 | 3.98 | 7.17 | 10 | 10 | 178 | 4 | 16 |
| A*24:02~B*07:02~DRB1*15:01 | 0.0086 | 5 | 1.14 | 0.67 | 9.71 | 13 | 13 | 337 | 183 | 72 |
| A*02:01~B*51:01~DRB1*11:01 | 0.0086 | 5 | 2.38 | 0.08 | 3.47 | 15 | 28 | 122 | 496 | 27 |
| A*01:01~B*08:01~DRB1*15:01 | 0.0086 | 5 | 8.38 | -0.23 | -0.17 | 25 | 25 | 544 | NA | NA |
| A*24:02~B*08:01~DRB1*03:01 | 0.0086 | 5 | 0.34 | 0.63 | 11.67 | 47 | 34 | 1379 | 268 | 42 |
| **A*02:01~B*15:01~DRB1*04:01** | 0.0069 | 4 | 2.22 | 2.53 | 7.06 | 9 | 6 | 21 | 1419 | 39 |
| A*02:01~B*18:01~DRB1*03:01 | 0.0069 | 4 | 0.22 | 0.17 | 2.82 | 68 | 71 | 80 | NA | 40 |
| A*02:01~B*40:01~DRB1*13:02 | 0.0052 | 3 | 1.11 | -0.35 | 3.16 | 7 | 9 | 81 | NA | 554 |
| A*02:01~B*08:01~DRB1*03:01 | 0.0052 | 3 | -1.86 | 0.17 | 11.67 | 17 | 11 | 20 | NA | 18 |
| A*02:01~B*18:01~DRB1*11:04 | 0.0052 | 3 | 0.22 | 0.08 | 5.69 | 24 | 33 | NA | NA | 70 |
| **A*31:01~B*40:01~DRB1*04:04** | 0.0052 | 3 | 5.40 | 4.09 | 6.07 | 27 | 20 | 98 | 1264 | 137 |
| A*02:01~B*51:01~DRB1*08:01 | 0.0052 | 3 | 2.38 | 0.55 | 1.78 | 29 | 73 | 245 | NA | 203 |
| A*32:01~B*44:03~DRB1*07:01 | 0.0052 | 3 | 2.06 | 1.81 | 9.25 | 120 | 291 | NA | 236 | 234 |
| **A*01:01~B*15:17~DRB1*13:02** | 0.0052 | 3 | 4.04 | 2.70 | 8.41 | 210 | 327 | NA | 55 | 284 |
| A*02:01~B*13:02~DRB1*13:01 | 0.0052 | 3 | -0.04 | 0.09 | 1.15 | 333 | NA | 1131 | NA | NA |
| A*02:01~B*13:02~DRB1*07:01 | 0.0034 | 2 | -0.04 | -1.10 | 7.17 | 11 | 19 | 183 | 132 | 131 |
| A*23:01~B*44:03~DRB1*07:01 | 0.0034 | 2 | 2.12 | 1.31 | 9.25 | 12 | 16 | 37 | NA | 7 |
| A*11:01~B*35:01~DRB1*01:01 | 0.0034 | 2 | 6.49 | 1.36 | 8.38 | 14 | 14 | 129 | 59 | 81 |
| A*02:01~B*57:01~DRB1*07:01 | 0.0034 | 2 | -0.91 | -1.10 | 5.85 | 16 | 12 | 680 | 123 | 135 |
| A*02:01~B*15:01~DRB1*13:01 | 0.0034 | 2 | 2.22 | 0.09 | 0.75 | 19 | 18 | 943 | NA | 373 |
| A*02:01~B*44:02~DRB1*11:01 | 0.0034 | 2 | 3.37 | 0.08 | 1.92 | 20 | 44 | 870 | 1397 | 85 |
| A*02:01~B*44:02~DRB1*13:01 | 0.0034 | 2 | 3.37 | 0.09 | 0.43 | 21 | 23 | 403 | NA | 15 |
| A*02:01~B*44:03~DRB1*07:01 | 0.0034 | 2 | -1.08 | -1.10 | 9.25 | 22 | 22 | 136 | 223 | 19 |
| A*01:01~B*07:02~DRB1*15:01 | 0.0034 | 2 | -1.87 | -0.23 | 9.71 | 23 | 17 | 172 | NA | 28 |
| A*02:01~B*51:01~DRB1*13:01 | 0.0034 | 2 | 2.38 | 0.09 | 1.26 | 30 | 39 | NA | 235 | 59 |
| **A*30:02~B*18:01~DRB1*03:01** | 0.0034 | 2 | 4.18 | 3.70 | 2.82 | 31 | 32 | 200 | NA | 4 |
| A*02:01~B*15:01~DRB1*15:01 | 0.0034 | 2 | 2.22 | 0.73 | -0.44 | 35 | 40 | 676 | NA | 222 |
| A*24:02~B*35:02~DRB1*11:04 | 0.0034 | 2 | 3.50 | 1.87 | 10.88 | 37 | 35 | 729 | 151 | 12 |
| A*02:01~B*44:02~DRB1*01:01 | 0.0034 | 2 | 3.37 | 0.18 | 1.31 | 39 | 24 | 461 | NA | 51 |
| A*02:01~B*51:01~DRB1*01:01 | 0.0034 | 2 | 2.38 | 0.18 | 0.57 | 40 | 125 | NA | NA | 123 |
| A*02:01~B*27:05~DRB1*01:01 | 0.0034 | 2 | 0.24 | 0.18 | 5.03 | 44 | 30 | 379 | 73 | 193 |
| A*03:01~B*07:02~DRB1*13:01 | 0.0034 | 2 | 3.93 | 1.31 | -0.84 | 45 | 46 | NA | NA | NA |
| A*24:02~B*51:01~DRB1*11:01 | 0.0034 | 2 | -0.32 | 2.03 | 3.47 | 46 | 93 | NA | 205 | NA |
| **A*68:02~B*14:02~DRB1*13:03** | 0.0034 | 2 | 8.38 | 4.62 | 3.71 | 48 | 38 | NA | NA | 1322 |
| A*02:01~B*51:01~DRB1*07:01 | 0.0034 | 2 | 2.38 | -1.10 | -0.46 | 49 | 80 | NA | 826 | 61 |
| A*03:01~B*15:01~DRB1*04:01 | 0.0034 | 2 | -0.09 | 1.14 | 7.06 | 57 | 58 | 336 | NA | NA |
| A*68:01~B*44:02~DRB1*11:01 | 0.0034 | 2 | 2.56 | 2.22 | 1.92 | 59 | 66 | 143 | NA | 351 |
| A*01:01~B*52:01~DRB1*15:02 | 0.0034 | 2 | 2.79 | 1.60 | 11.07 | 67 | 68 | 372 | 117 | 48 |
| A*03:01~B*35:03~DRB1*11:01 | 0.0034 | 2 | 1.39 | -0.71 | 2.88 | 88 | 1328 | NA | 175 | 467 |
| A*02:01~B*44:02~DRB1*16:01 | 0.0034 | 2 | 3.37 | 0.80 | 1.18 | 95 | 250 | NA | NA | NA |
| A*02:01~B*15:01~DRB1*14:54 | 0.0034 | 2 | 2.22 | -0.03 | 2.82 | 98 | 1438 | NA | 192 | NA |
| A*02:01~B*51:01~DRB1*15:01 | 0.0034 | 2 | 2.38 | 0.73 | -1.19 | 119 | 163 | NA | 210 | 88 |
| A*32:01~B*44:02~DRB1*11:01 | 0.0034 | 2 | 2.31 | 1.66 | 1.92 | 129 | 394 | NA | NA | NA |
| A*31:01~B*51:01~DRB1*13:01 | 0.0034 | 2 | 3.46 | 0.55 | 1.26 | 140 | NA | 1342 | NA | NA |
| A*68:01~B*51:01~DRB1*13:01 | 0.0034 | 2 | 3.01 | 0.73 | 1.26 | 149 | 116 | NA | NA | 612 |
| A*02:01~B*50:01~DRB1*03:01 | 0.0034 | 2 | 1.68 | 0.17 | 1.38 | 151 | 351 | NA | 788 | 149 |
| A*02:01~B*35:03~DRB1*11:01 | 0.0034 | 2 | 0.46 | 0.08 | 2.88 | 159 | 546 | NA | NA | 146 |
| A*03:01~B*35:01~DRB1*13:01 | 0.0034 | 2 | 4.31 | 1.31 | 0.49 | 162 | 147 | 1832 | NA | 44 |
| A*11:01~B*13:02~DRB1*07:01 | 0.0034 | 2 | 0.55 | 0.31 | 7.17 | 180 | 355 | NA | 456 | NA |
| A*32:01~B*40:02~DRB1*16:02 | 0.0034 | 2 | 1.97 | 2.98 | 4.84 | 204 | 681 | NA | NA | NA |
| A*01:01~B*49:01~DRB1*13:02 | 0.0034 | 2 | 0.45 | 2.70 | 2.87 | 215 | 1518 | NA | NA | 97 |
| **A*68:01~B*51:01~DRB1*11:01** | 0.0034 | 2 | 3.01 | 2.22 | 3.47 | 227 | 662 | NA | 366 | NA |
| A*02:01~B*50:01~DRB1*07:01 | 0.0034 | 2 | 1.68 | -1.10 | 2.54 | 230 | 233 | NA | 418 | 56 |
| A*01:01~B*57:01~DRB1*13:01 | 0.0034 | 2 | 6.78 | -0.52 | 0.32 | 274 | 234 | NA | NA | NA |
| A*02:01~B*38:01~DRB1*04:07 | 0.0034 | 2 | -1.16 | 0.94 | 3.24 | 331 | NA | NA | NA | NA |
| A*03:01~B*07:02~DRB1*04:03 | 0.0034 | 2 | 3.93 | 2.98 | 1.22 | 379 | NA | NA | NA | 1273 |
| A*26:01~B*18:01~DRB1*11:04 | 0.0034 | 2 | 0.95 | 0.96 | 5.69 | 438 | 339 | NA | NA | NA |
| A*26:01~B*07:02~DRB1*14:54 | 0.0034 | 2 | 0.39 | 2.35 | 1.01 | 456 | NA | NA | NA | NA |
| A*11:01~B*27:05~DRB1*01:01 | 0.0034 | 2 | 1.07 | 1.36 | 5.03 | 497 | 212 | 341 | 438 | 20 |
| A*68:01~B*07:02~DRB1*15:01 | 0.0034 | 2 | 0.30 | 1.10 | 9.71 | 512 | 174 | NA | 1256 | 343 |
| A*24:02~B*13:02~DRB1*11:01 | 0.0034 | 2 | 0.99 | 2.03 | 0.86 | 625 | NA | NA | NA | NA |
| A*02:01~B*49:01~DRB1*15:01 | 0.0034 | 2 | -0.15 | 0.73 | 0.10 | 674 | 695 | NA | NA | NA |
| A*26:01~B*38:01~DRB1*15:01 | 0.0034 | 2 | 9.71 | -0.75 | -0.32 | 681 | 856 | NA | NA | 742 |
| A*02:01~B*07:02~DRB1*01:03 | 0.0034 | 2 | 0.31 | 1.38 | 4.76 | NA | 504 | NA | NA | 90 |
| A*01:01~B*38:01~DRB1*14:01 | 0.0034 | 2 | -0.08 | 1.55 | 8.59 | NA | NA | NA | NA | NA |
| A*26:01~B*07:02~DRB1*13:02 | 0.0034 | 2 | 0.39 | 1.63 | -0.30 | NA | NA | NA | NA | NA |
| **A*68:01~B*40:02~DRB1*16:01** | 0.0034 | 2 | 3.00 | 2.33 | 2.75 | NA | NA | NA | NA | NA |
| A*01:01~B*41:02~DRB1*13:03 | 0.0034 | 2 | 1.92 | 0.93 | 9.81 | NA | NA | 736 | NA | NA |
| **A*34:01~B*38:02~DRB1*15:02** | 0.0034 | 2 | 24.04 | 11.12 | 11.12 | NA | NA | NA | 98 | NA |
| **A*33:01~B*14:02~DRB1*01:02** | 0.0017 | 1 | 7.27 | 4.66 | 9.10 | 26 | 15 | 427 | 1547 | 5 |
| A*25:01~B*18:01~DRB1*15:01 | 0.0017 | 1 | 1.32 | 1.95 | -0.29 | 33 | 21 | 38 | NA | 34 |
| A*01:01~B*08:01~DRB1*01:01 | 0.0017 | 1 | 8.38 | -0.68 | -1.34 | 38 | 45 | NA | NA | 609 |
| A*03:01~B*07:02~DRB1*01:01 | 0.0017 | 1 | 3.93 | 2.96 | -0.64 | 41 | 26 | 251 | 373 | 53 |
| A*03:01~B*51:01~DRB1*01:01 | 0.0017 | 1 | -1.49 | 2.96 | 0.57 | 50 | 83 | NA | NA | 321 |
| A*01:01~B*13:02~DRB1*07:01 | 0.0017 | 1 | -1.06 | 0.36 | 7.17 | 52 | 153 | 1927 | NA | 263 |
| A*11:01~B*52:01~DRB1*15:02 | 0.0017 | 1 | 0.62 | 1.77 | 11.07 | 53 | 62 | NA | 38 | 37 |
| A*11:01~B*35:01~DRB1*14:54 | 0.0017 | 1 | 6.49 | 0.87 | 0.22 | 54 | 149 | 1879 | NA | 106 |
| A*02:01~B*44:02~DRB1*15:01 | 0.0017 | 1 | 3.37 | 0.73 | 0.43 | 55 | 27 | 57 | NA | NA |
| A*24:02~B*13:02~DRB1*07:01 | 0.0017 | 1 | 0.99 | -0.24 | 7.17 | 56 | 86 | NA | 255 | 317 |
| A*24:02~B*18:01~DRB1*11:04 | 0.0017 | 1 | -0.51 | 1.87 | 5.69 | 61 | 64 | NA | NA | 153 |
| A*02:01~B*07:02~DRB1*01:01 | 0.0017 | 1 | 0.31 | 0.18 | -0.64 | 63 | 56 | NA | 325 | 108 |
| A*03:01~B*08:01~DRB1*03:01 | 0.0017 | 1 | -0.92 | -2.49 | 11.67 | 65 | 31 | 867 | NA | 58 |
| A*03:01~B*07:02~DRB1*04:04 | 0.0017 | 1 | 3.93 | -0.59 | 0.40 | 66 | 185 | 850 | NA | 1221 |
| A*11:01~B*51:01~DRB1*11:01 | 0.0017 | 1 | -0.34 | 0.73 | 3.47 | 70 | 353 | NA | 80 | 667 |
| A*03:01~B*35:01~DRB1*15:01 | 0.0017 | 1 | 4.31 | 2.11 | -1.21 | 75 | 137 | NA | NA | 348 |
| A*02:01~B*07:02~DRB1*11:01 | 0.0017 | 1 | 0.31 | 0.08 | -0.72 | 77 | 350 | 26 | NA | NA |
| A*02:01~B*38:01~DRB1*13:01 | 0.0017 | 1 | -1.16 | 0.09 | 4.56 | 79 | 78 | NA | NA | 621 |
| A*02:01~B*44:02~DRB1*11:04 | 0.0017 | 1 | 3.37 | 0.08 | 1.01 | 80 | 119 | 1443 | NA | 276 |
| A*01:01~B*57:01~DRB1*13:02 | 0.0017 | 1 | 6.78 | 2.70 | 0.22 | 81 | 641 | NA | NA | NA |
| A*01:01~B*37:01~DRB1*11:01 | 0.0017 | 1 | 4.44 | -1.60 | 0.95 | 83 | 188 | NA | 365 | NA |
| A*02:01~B*39:01~DRB1*01:01 | 0.0017 | 1 | 1.38 | 0.18 | 1.68 | 92 | 217 | NA | 836 | 541 |
| A*32:01~B*44:02~DRB1*12:01 | 0.0017 | 1 | 2.31 | 2.72 | 0.90 | 94 | 110 | NA | NA | NA |
| A*11:01~B*35:01~DRB1*11:01 | 0.0017 | 1 | 6.49 | 0.73 | -0.43 | 100 | 408 | NA | NA | 1196 |
| A*02:01~B*18:01~DRB1*11:01 | 0.0017 | 1 | 0.22 | 0.08 | -0.02 | 102 | 104 | 426 | NA | 366 |
| A*03:01~B*38:01~DRB1*13:01 | 0.0017 | 1 | 0.06 | 1.31 | 4.56 | 103 | 91 | 1398 | NA | 148 |
| A*11:01~B*08:01~DRB1*03:01 | 0.0017 | 1 | -1.29 | -0.13 | 11.67 | 104 | 145 | 307 | NA | 208 |
| A*23:01~B*49:01~DRB1*07:01 | 0.0017 | 1 | 5.91 | 1.31 | 0.06 | 106 | 670 | NA | NA | 1901 |
| A*02:01~B*44:02~DRB1*04:04 | 0.0017 | 1 | 3.37 | 0.24 | 0.31 | 108 | 127 | 2079 | NA | 717 |
| A*25:01~B*08:01~DRB1*03:01 | 0.0017 | 1 | 0.85 | 0.59 | 11.67 | 110 | 123 | 332 | NA | NA |
| A*24:02~B*35:01~DRB1*11:01 | 0.0017 | 1 | -0.32 | 2.03 | -0.43 | 113 | 522 | NA | 91 | NA |
| A*03:01~B*13:02~DRB1*07:01 | 0.0017 | 1 | -0.97 | -1.81 | 7.17 | 125 | 114 | NA | NA | 261 |
| A*01:01~B*08:01~DRB1*13:01 | 0.0017 | 1 | 8.38 | -0.52 | -1.48 | 128 | 54 | NA | NA | 89 |
| A*01:01~B*37:01~DRB1*10:01 | 0.0017 | 1 | 4.44 | 0.66 | 1.98 | 138 | 59 | 371 | 10 | 245 |
| A*02:01~B*51:01~DRB1*16:01 | 0.0017 | 1 | 2.38 | 0.80 | -0.49 | 146 | 521 | NA | NA | 1017 |
| A*24:02~B*15:01~DRB1*11:03 | 0.0017 | 1 | 1.94 | 1.10 | 4.35 | 148 | 143 | 1479 | NA | NA |
| A*24:02~B*35:01~DRB1*01:01 | 0.0017 | 1 | -0.32 | -0.82 | 8.38 | 152 | 206 | NA | NA | NA |
| A*01:01~B*37:01~DRB1*01:01 | 0.0017 | 1 | 4.44 | -0.68 | 0.24 | 160 | 198 | NA | NA | 884 |
| A*02:01~B*51:01~DRB1*04:01 | 0.0017 | 1 | 2.38 | 2.53 | 0.21 | 161 | 82 | NA | 679 | 1045 |
| A*01:01~B*08:01~DRB1*14:54 | 0.0017 | 1 | 8.38 | -0.15 | 0.51 | 165 | 373 | NA | NA | NA |
| A*02:01~B*41:02~DRB1*16:02 | 0.0017 | 1 | -0.03 | 0.15 | 4.29 | 169 | 1659 | 2011 | NA | NA |
| A*02:01~B*40:01~DRB1*15:01 | 0.0017 | 1 | 1.11 | 0.73 | 0.28 | 171 | 50 | 1985 | 321 | NA |
| A*02:01~B*07:02~DRB1*14:54 | 0.0017 | 1 | 0.31 | -0.03 | 1.01 | 172 | 133 | NA | NA | NA |
| A*02:01~B*15:01~DRB1*11:03 | 0.0017 | 1 | 2.22 | -0.03 | 4.35 | 187 | 667 | NA | NA | NA |
| A*01:01~B*15:01~DRB1*04:01 | 0.0017 | 1 | -1.32 | -0.94 | 7.06 | 193 | 108 | NA | NA | NA |
| A*03:01~B*35:03~DRB1*04:03 | 0.0017 | 1 | 1.39 | 2.98 | 1.64 | 195 | 489 | NA | 764 | 965 |
| A*31:01~B*51:01~DRB1*01:01 | 0.0017 | 1 | 3.46 | -0.19 | 0.57 | 200 | 676 | NA | 233 | 1121 |
| A*03:01~B*15:01~DRB1*13:01 | 0.0017 | 1 | -0.09 | 1.31 | 0.75 | 205 | 124 | NA | NA | 118 |
| A*11:01~B*35:01~DRB1*07:01 | 0.0017 | 1 | 6.49 | 0.31 | -1.25 | 213 | 170 | NA | 415 | 1418 |
| A*11:01~B*44:02~DRB1*13:01 | 0.0017 | 1 | -0.02 | 0.53 | 0.43 | 220 | 396 | NA | 702 | NA |
| A*03:01~B*50:01~DRB1*11:04 | 0.0017 | 1 | 1.16 | 1.03 | 1.60 | 225 | NA | NA | NA | NA |
| A*26:01~B*14:01~DRB1*07:01 | 0.0017 | 1 | 2.06 | -0.23 | 1.12 | 232 | 243 | NA | NA | 75 |
| A*24:02~B*18:01~DRB1*03:01 | 0.0017 | 1 | -0.51 | 0.63 | 2.82 | 236 | 543 | NA | NA | NA |
| A*02:01~B*44:02~DRB1*08:01 | 0.0017 | 1 | 3.37 | 0.55 | 0.47 | 237 | 115 | NA | NA | 660 |
| A*32:01~B*44:02~DRB1*11:04 | 0.0017 | 1 | 2.31 | 0.15 | 1.01 | 241 | 644 | 1845 | NA | 330 |
| A*02:01~B*14:02~DRB1*01:02 | 0.0017 | 1 | -0.97 | -0.43 | 9.10 | 243 | 77 | 359 | NA | 30 |
| A*33:03~B*58:01~DRB1*03:01 | 0.0017 | 1 | 2.34 | 0.41 | 2.16 | 247 | 985 | NA | 1 | NA |
| A*11:01~B*35:01~DRB1*03:01 | 0.0017 | 1 | 6.49 | -0.13 | -1.23 | 250 | 424 | NA | 880 | 239 |
| A*31:01~B*51:01~DRB1*04:07 | 0.0017 | 1 | 3.46 | 1.74 | 1.62 | 253 | 383 | NA | NA | NA |
| A*11:01~B*44:02~DRB1*15:01 | 0.0017 | 1 | -0.02 | -1.57 | 0.43 | 254 | 1132 | NA | NA | 1298 |
| A*01:01~B*51:01~DRB1*11:01 | 0.0017 | 1 | -2.02 | -1.60 | 3.47 | 256 | 470 | NA | NA | 553 |
| A*03:01~B*14:01~DRB1*04:01 | 0.0017 | 1 | 0.97 | 1.14 | 2.93 | 257 | NA | NA | NA | NA |
| A*02:01~B*07:02~DRB1*13:01 | 0.0017 | 1 | 0.31 | 0.09 | -0.84 | 259 | 94 | 797 | NA | 393 |
| A*03:01~B*35:01~DRB1*14:01 | 0.0017 | 1 | 4.31 | 1.68 | 1.48 | 261 | 247 | NA | NA | 617 |
| A*03:01~B*44:03~DRB1*07:01 | 0.0017 | 1 | -1.08 | -1.81 | 9.25 | 271 | 92 | 803 | NA | 113 |
| A*24:02~B*37:01~DRB1*07:01 | 0.0017 | 1 | 2.14 | -0.24 | -0.22 | 281 | 1236 | NA | NA | 1340 |
| **A*66:01~B*41:02~DRB1*13:03** | 0.0017 | 1 | 8.35 | 4.50 | 9.81 | 281 | 159 | NA | NA | 80 |
| A*03:01~B*18:01~DRB1*11:04 | 0.0017 | 1 | 0.39 | 1.03 | 5.69 | 290 | 141 | NA | 990 | 465 |
| A*01:01~B*08:01~DRB1*13:02 | 0.0017 | 1 | 8.38 | 2.70 | -0.70 | 293 | 279 | 578 | NA | NA |
| A*24:02~B*51:01~DRB1*07:01 | 0.0017 | 1 | -0.32 | -0.24 | -0.46 | 294 | 857 | NA | NA | 233 |
| A*32:01~B*27:05~DRB1*15:01 | 0.0017 | 1 | 0.46 | 0.13 | 0.37 | 296 | 308 | NA | NA | NA |
| A*03:01~B*40:02~DRB1*11:01 | 0.0017 | 1 | 0.37 | -0.71 | -0.12 | 298 | 632 | NA | NA | NA |
| A*11:01~B*35:03~DRB1*11:01 | 0.0017 | 1 | 1.89 | 0.73 | 2.88 | 305 | 1288 | NA | NA | 1432 |
| A*02:01~B*51:01~DRB1*11:02 | 0.0017 | 1 | 2.38 | 0.25 | 1.31 | 314 | 1837 | NA | NA | 542 |
| A*26:01~B*38:01~DRB1*07:01 | 0.0017 | 1 | 9.71 | -0.23 | -0.98 | 317 | 665 | NA | NA | NA |
| A*24:02~B*15:01~DRB1*11:01 | 0.0017 | 1 | 1.94 | 2.03 | -0.87 | 324 | 252 | NA | 61 | NA |
| A*03:01~B*14:02~DRB1*01:02 | 0.0017 | 1 | 1.55 | 0.26 | 9.10 | 330 | 106 | 737 | NA | 201 |
| A*02:01~B*35:02~DRB1*11:04 | 0.0017 | 1 | -0.43 | 0.08 | 10.88 | 333 | 184 | NA | NA | NA |
| A*02:01~B*07:02~DRB1*04:04 | 0.0017 | 1 | 0.31 | 0.24 | 0.40 | 337 | 112 | 436 | NA | NA |
| A*11:01~B*35:01~DRB1*16:01 | 0.0017 | 1 | 6.49 | -0.14 | 1.23 | 337 | 832 | NA | NA | NA |
| A*02:01~B*15:01~DRB1*03:01 | 0.0017 | 1 | 2.22 | 0.17 | -1.06 | 341 | 368 | NA | 254 | 672 |
| A*02:01~B*27:05~DRB1*03:01 | 0.0017 | 1 | 0.24 | 0.17 | 0.35 | 342 | 292 | NA | NA | NA |
| A*03:01~B*07:02~DRB1*16:01 | 0.0017 | 1 | 3.93 | 1.26 | -0.61 | 343 | 2343 | NA | NA | 180 |
| A*02:01~B*39:01~DRB1*12:01 | 0.0017 | 1 | 1.38 | -0.03 | 4.66 | 351 | 372 | NA | NA | NA |
| A*24:02~B*07:02~DRB1*11:01 | 0.0017 | 1 | 1.14 | 2.03 | -0.72 | 352 | 322 | NA | 732 | NA |
| A*02:01~B*56:01~DRB1*11:01 | 0.0017 | 1 | 0.37 | 0.08 | 1.91 | 357 | NA | NA | NA | NA |
| A*26:01~B*38:01~DRB1*14:01 | 0.0017 | 1 | 9.71 | 1.40 | 8.59 | 358 | 356 | NA | NA | NA |
| A*03:01~B*35:02~DRB1*11:04 | 0.0017 | 1 | 0.26 | 1.03 | 10.88 | 362 | 400 | NA | 966 | 320 |
| A*03:01~B*51:08~DRB1*11:04 | 0.0017 | 1 | 0.67 | 1.03 | 2.37 | 366 | NA | NA | NA | NA |
| A*32:01~B*14:01~DRB1*04:04 | 0.0017 | 1 | 2.32 | 0.46 | 3.39 | 366 | NA | NA | NA | NA |
| A*01:01~B*52:01~DRB1*04:04 | 0.0017 | 1 | 2.79 | 0.04 | 1.82 | 366 | NA | NA | NA | NA |
| A*01:01~B*57:01~DRB1*03:01 | 0.0017 | 1 | 6.78 | 4.08 | -0.82 | 372 | 1102 | NA | 446 | NA |
| A*03:01~B*35:03~DRB1*08:01 | 0.0017 | 1 | 1.39 | 0.37 | 1.15 | 373 | 619 | NA | NA | NA |
| A*26:01~B*44:03~DRB1*07:01 | 0.0017 | 1 | -0.17 | -0.23 | 9.25 | 376 | 319 | 1412 | NA | 527 |
| A*68:01~B*35:03~DRB1*04:01 | 0.0017 | 1 | 0.90 | 0.77 | 0.70 | 377 | 426 | NA | 1440 | NA |
| A*02:01~B*51:01~DRB1*10:01 | 0.0017 | 1 | 2.38 | -0.21 | 0.15 | 381 | 921 | NA | NA | NA |
| A*11:01~B*35:01~DRB1*10:01 | 0.0017 | 1 | 6.49 | 0.49 | 0.81 | 382 | 2148 | NA | NA | NA |
| A*01:01~B*44:02~DRB1*01:01 | 0.0017 | 1 | -1.05 | -0.68 | 1.31 | 383 | 401 | 1002 | NA | NA |
| A*03:01~B*27:05~DRB1*01:01 | 0.0017 | 1 | -0.59 | 2.96 | 5.03 | 384 | 209 | 550 | NA | 396 |
| A*32:01~B*51:01~DRB1*15:01 | 0.0017 | 1 | 0.36 | 0.13 | -1.19 | 386 | 627 | NA | 452 | 614 |
| A*01:01~B*35:01~DRB1*01:01 | 0.0017 | 1 | -1.50 | -0.68 | 8.38 | 389 | 118 | 1731 | NA | NA |
| A*03:01~B*18:01~DRB1*15:01 | 0.0017 | 1 | 0.39 | 2.11 | -0.29 | 393 | 205 | NA | NA | NA |
| A*11:01~B*18:01~DRB1*03:01 | 0.0017 | 1 | 1.21 | -0.13 | 2.82 | 397 | 162 | NA | NA | NA |
| A*02:01~B*07:10~DRB1*03:01 | 0.0017 | 1 | 1.47 | 0.17 | 2.67 | 397 | 1771 | NA | NA | NA |
| A*29:02~B*44:03~DRB1*14:54 | 0.0017 | 1 | 11.31 | 0.70 | 0.49 | 400 | 507 | NA | NA | NA |
| A*30:01~B*13:02~DRB1*11:01 | 0.0017 | 1 | 11.98 | 0.17 | 0.86 | 409 | 1077 | NA | 740 | NA |
| A*11:01~B*55:01~DRB1*11:01 | 0.0017 | 1 | 0.93 | 0.73 | 1.91 | 410 | NA | NA | NA | NA |
| A*11:01~B*35:01~DRB1*11:04 | 0.0017 | 1 | 6.49 | -0.25 | 0.05 | 412 | 756 | NA | NA | 266 |
| A*32:01~B*51:01~DRB1*12:01 | 0.0017 | 1 | 0.36 | 2.72 | 0.27 | 423 | NA | NA | 757 | NA |
| A*01:01~B*57:01~DRB1*14:54 | 0.0017 | 1 | 6.78 | -0.15 | 0.59 | 427 | 2229 | 1519 | NA | NA |
| A*02:01~B*08:01~DRB1*07:01 | 0.0017 | 1 | -1.86 | -1.10 | -1.52 | 428 | 617 | NA | NA | NA |
| A*02:01~B*41:01~DRB1*03:01 | 0.0017 | 1 | 0.25 | 0.17 | 1.14 | 430 | 2264 | NA | NA | NA |
| A*26:01~B*35:01~DRB1*01:01 | 0.0017 | 1 | 0.35 | -0.81 | 8.38 | 433 | 588 | NA | 747 | NA |
| A*02:01~B*58:01~DRB1*03:01 | 0.0017 | 1 | -0.73 | 0.17 | 2.16 | 434 | NA | NA | NA | 249 |
| A*24:02~B*35:02~DRB1*11:01 | 0.0017 | 1 | 3.50 | 2.03 | 0.58 | 442 | NA | NA | NA | NA |
| A*03:01~B*40:02~DRB1*13:01 | 0.0017 | 1 | 0.37 | 1.31 | 2.13 | 449 | 723 | NA | NA | NA |
| A*02:01~B*52:01~DRB1*15:02 | 0.0017 | 1 | -0.73 | -0.86 | 11.07 | 450 | 72 | NA | NA | 114 |
| A*02:01~B*51:01~DRB1*08:03 | 0.0017 | 1 | 2.38 | -0.25 | 0.75 | 452 | 421 | NA | NA | NA |
| A*11:01~B*51:01~DRB1*01:01 | 0.0017 | 1 | -0.34 | 1.36 | 0.57 | 456 | 266 | NA | NA | 604 |
| A*26:01~B*27:05~DRB1*04:04 | 0.0017 | 1 | 0.28 | 0.28 | 1.12 | 477 | 981 | NA | 453 | 584 |
| **A*26:01~B*55:01~DRB1*14:54** | 0.0017 | 1 | 4.79 | 2.35 | 4.47 | 478 | 277 | NA | NA | NA |
| A*03:01~B*55:01~DRB1*14:54 | 0.0017 | 1 | 0.26 | -0.73 | 4.47 | 482 | 1232 | NA | NA | NA |
| A*03:01~B*51:01~DRB1*13:02 | 0.0017 | 1 | -1.49 | -0.55 | -0.80 | 483 | NA | NA | NA | 1751 |
| A*01:01~B*37:01~DRB1*13:02 | 0.0017 | 1 | 4.44 | 2.70 | 0.84 | 494 | 1184 | NA | NA | 410 |
| A*24:02~B*27:05~DRB1*01:01 | 0.0017 | 1 | -0.20 | -0.82 | 5.03 | 494 | 237 | NA | NA | NA |
| **A*30:04~B*51:08~DRB1*13:02** | 0.0017 | 1 | 6.70 | 2.52 | 2.09 | 497 | 1726 | NA | NA | NA |
| **A*31:01~B*51:01~DRB1*04:08** | 0.0017 | 1 | 3.46 | 2.55 | 2.60 | 497 | 1165 | NA | NA | 183 |
| A*68:01~B*44:02~DRB1*15:01 | 0.0017 | 1 | 2.56 | 1.10 | 0.43 | 498 | 345 | NA | NA | NA |
| A*02:01~B*18:01~DRB1*01:01 | 0.0017 | 1 | 0.22 | 0.18 | -0.87 | 499 | 637 | 2029 | NA | NA |
| A*31:01~B*13:02~DRB1*07:01 | 0.0017 | 1 | 0.53 | 0.82 | 7.17 | 504 | NA | NA | NA | 1734 |
| A*32:01~B*50:01~DRB1*07:01 | 0.0017 | 1 | 1.21 | 1.81 | 2.54 | 507 | 843 | NA | NA | NA |
| A*01:01~B*40:01~DRB1*15:01 | 0.0017 | 1 | -0.75 | -0.23 | 0.28 | 510 | 969 | NA | NA | 506 |
| A*29:02~B*08:01~DRB1*03:01 | 0.0017 | 1 | -0.45 | -0.70 | 11.67 | 512 | 255 | NA | NA | NA |
| A*26:01~B*55:01~DRB1*11:01 | 0.0017 | 1 | 4.79 | -0.53 | 1.91 | 514 | 845 | NA | NA | NA |
| A*02:01~B*40:01~DRB1*04:08 | 0.0017 | 1 | 1.11 | -0.03 | 2.75 | 516 | NA | NA | NA | 470 |
| A*11:01~B*35:01~DRB1*08:01 | 0.0017 | 1 | 6.49 | 0.21 | 0.51 | 520 | 502 | NA | NA | NA |
| A*11:01~B*40:01~DRB1*13:01 | 0.0017 | 1 | -0.02 | 0.53 | -0.18 | 529 | 1373 | NA | NA | NA |
| A*24:02~B*49:01~DRB1*13:02 | 0.0017 | 1 | -0.39 | -0.70 | 2.87 | 531 | 951 | NA | 953 | NA |
| A*30:01~B*13:02~DRB1*16:01 | 0.0017 | 1 | 11.98 | 1.41 | 0.53 | 534 | NA | NA | NA | NA |
| A*03:01~B*56:01~DRB1*01:01 | 0.0017 | 1 | 0.26 | 2.96 | 0.87 | 535 | 333 | NA | NA | 992 |
| A*02:01~B*40:02~DRB1*13:01 | 0.0017 | 1 | -0.01 | 0.09 | 2.13 | 535 | 361 | NA | NA | NA |
| A*29:02~B*44:03~DRB1*04:01 | 0.0017 | 1 | 11.31 | 1.91 | 0.38 | 536 | 446 | 477 | 1517 | NA |
| A*01:01~B*08:01~DRB1*04:03 | 0.0017 | 1 | 8.38 | 0.84 | 1.56 | 543 | NA | NA | NA | NA |
| A*02:01~B*37:01~DRB1*11:01 | 0.0017 | 1 | -1.08 | 0.08 | 0.95 | 547 | 961 | 353 | 1444 | NA |
| A*24:02~B*15:01~DRB1*14:54 | 0.0017 | 1 | 1.94 | -0.33 | 2.82 | 553 | 313 | NA | NA | NA |
| A*29:02~B*51:01~DRB1*11:01 | 0.0017 | 1 | -0.54 | -0.53 | 3.47 | 555 | NA | NA | NA | NA |
| A*26:01~B*55:01~DRB1*13:02 | 0.0017 | 1 | 4.79 | 1.63 | 1.55 | 575 | NA | NA | NA | NA |
| A*02:01~B*39:06~DRB1*04:04 | 0.0017 | 1 | 1.38 | 0.24 | 3.39 | 578 | NA | NA | NA | NA |
| A*02:01~B*53:01~DRB1*13:02 | 0.0017 | 1 | -0.43 | -0.35 | 3.58 | 578 | 1999 | NA | NA | 825 |
| A*02:01~B*49:01~DRB1*13:03 | 0.0017 | 1 | -0.15 | 0.39 | 2.60 | 578 | 2217 | 554 | NA | NA |
| A*02:01~B*35:08~DRB1*13:03 | 0.0017 | 1 | 0.37 | 0.39 | 2.34 | 578 | NA | NA | NA | NA |
| A*02:01~B*58:01~DRB1*10:01 | 0.0017 | 1 | -0.73 | -0.21 | 6.30 | 578 | NA | NA | NA | 767 |
| **A*30:02~B*41:01~DRB1*04:05** | 0.0017 | 1 | 5.00 | 2.89 | 4.66 | 578 | 1635 | NA | NA | NA |
| A*01:01~B*57:01~DRB1*04:02 | 0.0017 | 1 | 6.78 | 0.36 | 1.97 | 583 | 561 | NA | NA | 677 |
| A*24:02~B*35:03~DRB1*07:01 | 0.0017 | 1 | -0.33 | -0.24 | -0.60 | 590 | 483 | 1705 | NA | 1321 |
| A*68:01~B*51:01~DRB1*07:01 | 0.0017 | 1 | 3.01 | -0.53 | -0.46 | 591 | 1535 | NA | NA | 170 |
| A*25:01~B*44:02~DRB1*01:01 | 0.0017 | 1 | 1.44 | 1.07 | 1.31 | 601 | 397 | NA | NA | NA |
| A*33:01~B*14:02~DRB1*07:01 | 0.0017 | 1 | 7.27 | 0.81 | -0.12 | 612 | 776 | 498 | 1467 | 1604 |
| A*11:01~B*35:03~DRB1*04:08 | 0.0017 | 1 | 1.89 | 3.30 | 2.64 | 616 | 1269 | NA | NA | 313 |
| A*32:01~B*07:02~DRB1*11:01 | 0.0017 | 1 | -0.47 | 1.66 | -0.72 | 617 | NA | 356 | NA | 344 |
| A*02:01~B*35:03~DRB1*12:01 | 0.0017 | 1 | 0.46 | -0.03 | 1.64 | 625 | 107 | NA | NA | NA |
| **A*03:02~B*55:01~DRB1*12:01** | 0.0017 | 1 | 6.79 | 5.84 | 3.17 | 627 | NA | NA | NA | NA |
| A*32:01~B*18:01~DRB1*14:54 | 0.0017 | 1 | -0.40 | 0.32 | 0.09 | 630 | NA | NA | NA | NA |
| A*24:02~B*35:08~DRB1*04:03 | 0.0017 | 1 | 2.04 | 0.37 | 3.17 | 630 | 493 | NA | NA | 1778 |
| A*24:02~B*39:01~DRB1*11:01 | 0.0017 | 1 | 1.43 | 2.03 | 1.34 | 631 | NA | NA | NA | NA |
| A*03:01~B*27:02~DRB1*16:01 | 0.0017 | 1 | 0.97 | 1.26 | 6.36 | 639 | 1506 | NA | NA | 1660 |
| A*02:01~B*08:01~DRB1*11:01 | 0.0017 | 1 | -1.86 | 0.08 | -1.69 | 642 | NA | 1213 | NA | 997 |
| A*01:01~B*18:01~DRB1*04:03 | 0.0017 | 1 | -1.66 | 0.84 | 0.80 | 643 | 719 | NA | NA | NA |
| A*03:01~B*38:01~DRB1*16:01 | 0.0017 | 1 | 0.06 | 1.26 | 0.39 | 646 | NA | NA | NA | NA |
| A*02:01~B*44:03~DRB1*03:01 | 0.0017 | 1 | -1.08 | 0.17 | -0.92 | 651 | 542 | NA | NA | NA |
| A*26:01~B*38:01~DRB1*08:01 | 0.0017 | 1 | 9.71 | 0.44 | 0.75 | 653 | NA | NA | NA | NA |
| A*29:02~B*38:01~DRB1*01:01 | 0.0017 | 1 | 0.33 | 0.63 | -0.49 | 655 | NA | NA | NA | NA |
| A*02:01~B*51:01~DRB1*04:02 | 0.0017 | 1 | 2.38 | -0.25 | 2.19 | 656 | 933 | NA | 1361 | NA |
| A*30:02~B*18:01~DRB1*15:01 | 0.0017 | 1 | 4.18 | 0.27 | -0.29 | 658 | 1520 | 1877 | NA | 1592 |
| A*01:01~B*38:01~DRB1*03:01 | 0.0017 | 1 | -0.08 | 4.08 | -0.97 | 660 | NA | NA | NA | NA |
| A*30:01~B*38:01~DRB1*12:01 | 0.0017 | 1 | 1.06 | 2.46 | 1.20 | 660 | NA | NA | NA | NA |
| A*24:02~B*35:43~DRB1*04:07 | 0.0017 | 1 | 3.10 | 1.78 | 8.97 | 660 | NA | 1171 | NA | 41 |
| A*01:01~B*27:05~DRB1*09:01 | 0.0017 | 1 | -0.68 | 0.59 | 6.05 | 660 | 821 | NA | NA | NA |
| A*24:02~B*07:02~DRB1*01:03 | 0.0017 | 1 | 1.14 | 1.43 | 4.76 | 660 | 1122 | NA | NA | 1146 |
| A*30:02~B*53:01~DRB1*13:02 | 0.0017 | 1 | 3.42 | 1.36 | 3.58 | 660 | 1629 | NA | 1296 | 834 |
| A*01:01~B*57:01~DRB1*04:04 | 0.0017 | 1 | 6.78 | 0.04 | 0.73 | 678 | 480 | NA | NA | 412 |
| A*11:01~B*18:01~DRB1*07:01 | 0.0017 | 1 | 1.21 | 0.31 | -1.40 | 703 | 554 | NA | NA | 1122 |
| A*03:01~B*14:02~DRB1*04:05 | 0.0017 | 1 | 1.55 | -0.02 | 2.30 | 705 | NA | NA | NA | 548 |
| A*32:01~B*08:01~DRB1*03:01 | 0.0017 | 1 | -0.83 | -1.10 | 11.67 | 715 | 168 | NA | 481 | NA |
| A*24:02~B*15:01~DRB1*12:02 | 0.0017 | 1 | 1.94 | 1.10 | 1.97 | 720 | NA | NA | 370 | NA |
| A*11:01~B*15:02~DRB1*12:02 | 0.0017 | 1 | 3.59 | 1.39 | 11.89 | 720 | NA | 2138 | 6 | NA |
| A*24:02~B*44:05~DRB1*07:01 | 0.0017 | 1 | 3.10 | -0.24 | 2.64 | 733 | NA | NA | NA | NA |
| A*01:01~B*44:02~DRB1*07:01 | 0.0017 | 1 | -1.05 | 0.36 | -1.29 | 736 | 2419 | NA | NA | NA |
| A*02:03~B*52:01~DRB1*14:54 | 0.0017 | 1 | 8.38 | 5.83 | 1.64 | NA | NA | NA | NA | NA |
| A*68:01~B*35:02~DRB1*11:04 | 0.0017 | 1 | 2.12 | 0.71 | 10.88 | NA | 1240 | NA | NA | NA |
| A*02:01~B*07:06~DRB1*16:01 | 0.0017 | 1 | 0.25 | 0.80 | 3.03 | NA | NA | NA | NA | NA |
| A*30:02~B*08:01~DRB1*03:01 | 0.0017 | 1 | 0.50 | 3.70 | 11.67 | NA | 464 | 86 | NA | NA |
| **A*23:01~B*58:01~DRB1*09:01** | 0.0017 | 1 | 3.14 | 3.01 | 3.01 | NA | NA | 1262 | NA | NA |
| A*24:02~B*18:03~DRB1*11:04 | 0.0017 | 1 | 3.10 | 1.87 | 5.33 | NA | NA | NA | NA | NA |
| A*26:01~B*38:01~DRB1*08:03 | 0.0017 | 1 | 9.71 | 1.40 | 1.78 | NA | 878 | NA | 1364 | 1575 |
| A*32:01~B*35:03~DRB1*15:01 | 0.0017 | 1 | 0.32 | 0.13 | -0.56 | NA | 2249 | NA | NA | NA |
| A*02:01~B*57:03~DRB1*13:02 | 0.0017 | 1 | 0.69 | -0.35 | 3.26 | NA | NA | NA | NA | NA |
| A*68:02~B*15:10~DRB1*03:01 | 0.0017 | 1 | 5.00 | 1.38 | 2.85 | NA | NA | 4 | NA | 121 |
| A*03:01~B*50:01~DRB1*13:01 | 0.0017 | 1 | 1.16 | 1.31 | 0.59 | NA | NA | NA | NA | NA |
| **A*02:02~B*15:03~DRB1*13:01** | 0.0017 | 1 | 17.00 | 2.11 | 3.26 | NA | NA | NA | NA | NA |
| A*29:02~B*07:02~DRB1*15:03 | 0.0017 | 1 | 0.05 | 2.46 | 0.86 | NA | NA | NA | NA | NA |
| A*11:12~B*18:01~DRB1*11:01 | 0.0017 | 1 | 3.94 | 2.96 | -0.02 | NA | NA | NA | NA | NA |
| A*25:01~B*57:01~DRB1*15:01 | 0.0017 | 1 | 1.97 | 1.95 | -0.80 | NA | 870 | NA | NA | NA |
| A*03:01~B*15:10~DRB1*13:02 | 0.0017 | 1 | 0.97 | -0.55 | 2.52 | NA | NA | NA | NA | NA |
| A*29:02~B*07:02~DRB1*08:04 | 0.0017 | 1 | 0.05 | 1.87 | 0.44 | NA | NA | NA | NA | NA |
| A*02:02~B*08:01~DRB1*15:03 | 0.0017 | 1 | 1.99 | 8.35 | 1.10 | NA | NA | NA | NA | NA |
| A*02:05~B*51:01~DRB1*04:05 | 0.0017 | 1 | 1.31 | 4.66 | 0.27 | NA | NA | NA | NA | NA |
| A*11:01~B*57:01~DRB1*07:01 | 0.0017 | 1 | -0.30 | 0.31 | 5.85 | NA | 614 | NA | 217 | 1902 |
| **A*23:01~B*58:01~DRB1*13:04** | 0.0017 | 1 | 3.14 | 6.51 | 6.51 | NA | NA | NA | NA | NA |
| A*68:01~B*27:05~DRB1*09:01 | 0.0017 | 1 | 1.04 | 2.75 | 6.05 | NA | NA | NA | NA | NA |
| A*03:01~B*18:01~DRB1*10:01 | 0.0017 | 1 | 0.39 | 0.78 | 0.67 | NA | NA | NA | NA | NA |
| A*03:01~B*58:01~DRB1*10:01 | 0.0017 | 1 | -0.51 | 0.78 | 6.30 | NA | NA | NA | NA | NA |
| A*24:02~B*40:02~DRB1*15:01 | 0.0017 | 1 | -0.04 | 0.67 | -0.27 | NA | NA | NA | 52 | NA |
| A*23:01~B*49:01~DRB1*03:01 | 0.0017 | 1 | 5.91 | -0.37 | 0.08 | NA | 659 | NA | NA | NA |
| A*24:02~B*07:05~DRB1*04:07 | 0.0017 | 1 | 1.99 | 1.78 | 6.27 | NA | NA | NA | NA | NA |
| A*31:01~B*56:01~DRB1*08:01 | 0.0017 | 1 | 4.31 | 1.08 | 2.46 | NA | NA | NA | NA | NA |
| A*02:01~B*40:01~DRB1*13:03 | 0.0017 | 1 | 1.11 | 0.39 | 1.13 | NA | NA | NA | NA | NA |
| A*24:02~B*37:01~DRB1*08:01 | 0.0017 | 1 | 2.14 | 0.93 | 3.68 | NA | 1273 | NA | NA | NA |
| A*01:01~B*35:08~DRB1*10:01 | 0.0017 | 1 | 0.18 | 0.66 | 2.94 | NA | 742 | NA | NA | NA |
| **A*29:01~B*07:05~DRB1*04:02** | 0.0017 | 1 | 17.00 | 10.63 | 7.45 | NA | NA | NA | NA | 282 |
| A*02:01~B*08:01~DRB1*14:54 | 0.0017 | 1 | -1.86 | -0.03 | 0.51 | NA | NA | 981 | NA | 885 |
| **A*23:01~B*49:01~DRB1*08:03** | 0.0017 | 1 | 5.91 | 2.63 | 2.21 | NA | NA | NA | NA | NA |
| **A*30:02~B*27:03~DRB1*03:01** | 0.0017 | 1 | 8.97 | 3.70 | 2.67 | NA | NA | NA | NA | NA |
| A*01:01~B*07:02~DRB1*11:01 | 0.0017 | 1 | -1.87 | -1.60 | -0.72 | NA | 705 | NA | NA | NA |
| A*29:02~B*40:02~DRB1*01:01 | 0.0017 | 1 | 1.02 | 0.63 | 0.16 | NA | NA | NA | NA | NA |
| A*01:01~B*07:06~DRB1*07:01 | 0.0017 | 1 | 0.89 | 0.36 | 1.12 | NA | NA | 512 | NA | NA |
| A*31:01~B*40:01~DRB1*01:02 | 0.0017 | 1 | 5.40 | 1.95 | 2.12 | NA | NA | NA | NA | NA |
| A*24:02~B*07:02~DRB1*08:03 | 0.0017 | 1 | 1.14 | 2.36 | 0.62 | NA | NA | NA | NA | NA |
| A*03:01~B*14:02~DRB1*08:01 | 0.0017 | 1 | 1.55 | 0.37 | 1.72 | NA | NA | NA | NA | NA |
| A*01:01~B*37:01~DRB1*13:03 | 0.0017 | 1 | 4.44 | 0.93 | 1.50 | NA | NA | NA | NA | NA |
| A*02:01~B*39:06~DRB1*01:01 | 0.0017 | 1 | 1.38 | 0.18 | 1.68 | NA | 1366 | 705 | NA | NA |
| **A*24:33~B*51:06~DRB1*09:01** | 0.0017 | 1 | 24.08 | 11.89 | 11.89 | NA | NA | NA | NA | NA |
| A*25:01~B*07:02~DRB1*12:01 | 0.0017 | 1 | 0.62 | 3.52 | 0.15 | NA | NA | NA | NA | NA |
| A*23:01~B*40:01~DRB1*04:04 | 0.0017 | 1 | 1.13 | 1.21 | 6.07 | NA | NA | NA | NA | NA |
| A*03:20~B*07:02~DRB1*15:01 | 0.0017 | 1 | 2.72 | 2.70 | 9.71 | NA | NA | NA | NA | NA |
| A*33:03~B*57:01~DRB1*07:01 | 0.0017 | 1 | 1.72 | 0.39 | 5.85 | NA | NA | NA | 611 | NA |
| A*03:01~B*51:01~DRB1*04:01 | 0.0017 | 1 | -1.49 | 1.14 | 0.21 | NA | 1279 | NA | 1201 | NA |
| A*24:02~B*35:08~DRB1*01:02 | 0.0017 | 1 | 2.04 | 0.66 | 3.73 | NA | NA | NA | NA | NA |
| A*03:01~B*45:01~DRB1*11:01 | 0.0017 | 1 | 1.47 | -0.71 | 1.88 | NA | NA | NA | NA | NA |
| **A*02:05~B*58:01~DRB1*08:04** | 0.0017 | 1 | 3.55 | 5.43 | 2.34 | NA | NA | NA | NA | NA |
| A*02:01~B*56:01~DRB1*04:04 | 0.0017 | 1 | 0.37 | 0.24 | 2.22 | NA | NA | NA | NA | NA |
| A*32:01~B*39:06~DRB1*13:02 | 0.0017 | 1 | 2.32 | -0.04 | 2.52 | NA | NA | NA | NA | NA |
| A*02:20~B*51:01~DRB1*13:01 | 0.0017 | 1 | 2.93 | 3.26 | 1.26 | NA | NA | NA | NA | NA |
| A*24:02~B*15:01~DRB1*08:01 | 0.0017 | 1 | 1.94 | 0.93 | 0.66 | NA | 2444 | NA | NA | NA |
| A*31:01~B*44:03~DRB1*07:01 | 0.0017 | 1 | 1.67 | 0.82 | 9.25 | NA | 1116 | NA | NA | 362 |
| A*03:01~B*08:01~DRB1*07:01 | 0.0017 | 1 | -0.92 | -1.81 | -1.52 | NA | NA | NA | NA | NA |
| A*26:01~B*51:01~DRB1*03:01 | 0.0017 | 1 | -1.13 | -0.77 | -2.05 | NA | NA | NA | NA | NA |
| A*68:02~B*18:01~DRB1*03:01 | 0.0017 | 1 | 0.94 | 1.38 | 2.82 | NA | 1991 | 456 | NA | 264 |
| A*29:02~B*44:03~DRB1*04:05 | 0.0017 | 1 | 11.31 | 1.50 | 1.25 | NA | NA | NA | NA | 712 |
| **A*01:03~B*49:01~DRB1*13:03** | 0.0017 | 1 | 5.65 | 6.51 | 2.60 | NA | NA | NA | NA | NA |
| A*11:01~B*15:01~DRB1*04:06 | 0.0017 | 1 | -0.53 | 2.35 | 3.10 | NA | NA | NA | 23 | NA |
| A*23:01~B*18:01~DRB1*11:01 | 0.0017 | 1 | 0.30 | -0.20 | -0.02 | NA | NA | NA | NA | 454 |
| A*01:01~B*49:01~DRB1*13:01 | 0.0017 | 1 | 0.45 | -0.52 | -0.30 | NA | 2319 | NA | NA | NA |
| A*24:02~B*51:01~DRB1*04:02 | 0.0017 | 1 | -0.32 | 2.36 | 2.19 | NA | NA | NA | NA | NA |
| **A*30:04~B*58:02~DRB1*15:03** | 0.0017 | 1 | 13.72 | 6.70 | 11.89 | NA | NA | NA | NA | NA |
| A*11:01~B*51:01~DRB1*04:08 | 0.0017 | 1 | -0.34 | 3.30 | 2.60 | NA | 1801 | 1515 | NA | NA |
| A*32:01~B*07:02~DRB1*01:01 | 0.0017 | 1 | -0.47 | 0.11 | -0.64 | NA | 694 | 675 | NA | NA |
| **A*02:131~B*07:06~DRB1*08:02** | 0.0017 | 1 | 13.72 | 24.08 | 13.72 | NA | NA | NA | NA | NA |
| **A*24:02~B*37:01~DRB1*08:03** | 0.0017 | 1 | 2.14 | 2.36 | 2.91 | NA | NA | NA | NA | NA |
| A*02:01~B*27:05~DRB1*15:01 | 0.0017 | 1 | 0.24 | 0.73 | 0.37 | NA | 98 | 1172 | NA | NA |
| A*26:01~B*56:01~DRB1*11:01 | 0.0017 | 1 | 1.18 | -0.53 | 1.91 | NA | NA | NA | NA | NA |
| **A*01:01~B*37:01~DRB1*14:07** | 0.0017 | 1 | 4.44 | 2.31 | 7.10 | NA | NA | NA | NA | NA |
| A*32:01~B*40:01~DRB1*10:01 | 0.0017 | 1 | 0.39 | 0.92 | 1.57 | NA | 2304 | NA | NA | NA |
| A*01:01~B*51:01~DRB1*15:02 | 0.0017 | 1 | -2.02 | 1.60 | 0.15 | NA | NA | NA | NA | NA |
| A*24:02~B*35:14~DRB1*16:02 | 0.0017 | 1 | 3.10 | 0.50 | 8.97 | NA | NA | NA | NA | 329 |
| A*31:01~B*27:05~DRB1*03:01 | 0.0017 | 1 | 0.91 | -0.64 | 0.35 | NA | NA | NA | NA | NA |
| A*31:01~B*56:01~DRB1*15:01 | 0.0017 | 1 | 4.31 | -0.63 | 0.42 | NA | NA | NA | NA | NA |
| A*01:01~B*52:01~DRB1*13:01 | 0.0017 | 1 | 2.79 | -0.52 | 0.45 | NA | NA | NA | NA | NA |
| A*11:01~B*35:03~DRB1*14:54 | 0.0017 | 1 | 1.89 | 0.87 | 0.83 | NA | NA | NA | NA | 962 |
| A*26:01~B*35:01~DRB1*16:01 | 0.0017 | 1 | 0.35 | 1.13 | 1.23 | NA | NA | NA | NA | NA |
| A*30:04~B*51:01~DRB1*04:03 | 0.0017 | 1 | 1.31 | 4.66 | 0.27 | NA | NA | NA | NA | NA |
| A*32:01~B*49:01~DRB1*07:01 | 0.0017 | 1 | 0.26 | 1.81 | 0.06 | NA | NA | 1243 | NA | NA |
| A*01:01~B*37:01~DRB1*08:01 | 0.0017 | 1 | 4.44 | -0.51 | 3.68 | NA | 1492 | NA | NA | NA |
| A*02:01~B*40:02~DRB1*14:05 | 0.0017 | 1 | -0.01 | 1.47 | 6.79 | NA | NA | NA | NA | NA |
| A*23:01~B*45:01~DRB1*13:01 | 0.0017 | 1 | 4.50 | -0.04 | 2.11 | NA | NA | 125 | NA | 633 |
| A*11:01~B*47:01~DRB1*11:01 | 0.0017 | 1 | 2.35 | 0.73 | 1.88 | NA | NA | NA | NA | NA |
| **A*02:06~B*15:01~DRB1*04:10** | 0.0017 | 1 | 3.10 | 17.00 | 4.59 | NA | NA | NA | NA | NA |
| A*23:01~B*07:02~DRB1*11:04 | 0.0017 | 1 | -0.33 | 0.87 | -0.72 | NA | NA | NA | NA | NA |
| A*03:01~B*35:08~DRB1*11:01 | 0.0017 | 1 | 0.26 | -0.71 | 0.58 | NA | NA | NA | NA | NA |
| **A*33:01~B*50:02~DRB1*04:06** | 0.0017 | 1 | 11.89 | 8.35 | 17.00 | NA | NA | NA | NA | NA |
| A*32:01~B*27:02~DRB1*11:01 | 0.0017 | 1 | 2.32 | 1.66 | 1.34 | NA | 2426 | NA | NA | NA |
| A*02:01~B*51:08~DRB1*16:01 | 0.0017 | 1 | 0.95 | 0.80 | 2.55 | NA | NA | NA | NA | NA |
| A*11:01~B*35:01~DRB1*13:01 | 0.0017 | 1 | 6.49 | 0.53 | 0.49 | NA | 218 | 633 | 135 | 1740 |
| A*32:01~B*35:01~DRB1*01:01 | 0.0017 | 1 | -0.27 | 0.11 | 8.38 | NA | 321 | 1531 | NA | NA |
| A*03:01~B*08:01~DRB1*11:02 | 0.0017 | 1 | -0.92 | 0.97 | 1.43 | NA | NA | NA | NA | NA |
| A*33:03~B*18:01~DRB1*13:03 | 0.0017 | 1 | 1.11 | 2.34 | 0.30 | NA | NA | NA | NA | NA |
| A*31:01~B*51:01~DRB1*07:01 | 0.0017 | 1 | 3.46 | 0.82 | -0.46 | NA | NA | NA | 793 | NA |
| A*26:09~B*51:01~DRB1*04:07 | 0.0017 | 1 | 2.93 | 8.97 | 1.62 | NA | NA | NA | NA | NA |
| A*02:01~B*51:08~DRB1*16:02 | 0.0017 | 1 | 0.95 | 0.15 | 4.29 | NA | NA | NA | NA | 1817 |
| A*26:01~B*40:01~DRB1*11:03 | 0.0017 | 1 | 0.21 | 1.68 | 2.75 | NA | 1606 | NA | NA | NA |
| A*02:01~B*44:27~DRB1*16:01 | 0.0017 | 1 | 1.47 | 0.80 | 5.65 | NA | NA | NA | NA | NA |
| **A*23:01~B*49:01~DRB1*11:03** | 0.0017 | 1 | 5.91 | 3.01 | 2.55 | NA | 1908 | NA | NA | NA |
| A*11:01~B*07:02~DRB1*03:01 | 0.0017 | 1 | -1.06 | -0.13 | -2.22 | NA | 606 | NA | NA | NA |
| A*03:02~B*35:03~DRB1*15:02 | 0.0017 | 1 | 4.01 | 5.47 | 1.49 | NA | NA | NA | NA | NA |
| A*31:01~B*44:03~DRB1*12:01 | 0.0017 | 1 | 1.67 | 1.57 | 1.25 | NA | NA | NA | NA | NA |
| **A*66:02~B*58:01~DRB1*15:03** | 0.0017 | 1 | 6.51 | 11.89 | 3.01 | NA | NA | 22 | NA | 279 |
| A*03:01~B*18:01~DRB1*13:01 | 0.0017 | 1 | 0.39 | 1.31 | -0.39 | NA | 476 | NA | NA | 1188 |
| A*66:01~B*58:01~DRB1*13:01 | 0.0017 | 1 | 4.50 | 2.11 | -0.04 | NA | NA | NA | NA | NA |
| **A*33:03~B*15:16~DRB1*01:02** | 0.0017 | 1 | 9.70 | 3.73 | 9.70 | NA | NA | 55 | NA | NA |
| A*02:01~B*35:03~DRB1*01:01 | 0.0017 | 1 | 0.46 | 0.18 | -0.13 | NA | NA | NA | NA | NA |
| A*26:01~B*18:01~DRB1*16:01 | 0.0017 | 1 | 0.95 | 1.13 | 0.03 | NA | NA | NA | NA | NA |
| **A*68:02~B*14:02~DRB1*11:02** | 0.0017 | 1 | 8.38 | 5.00 | 4.11 | NA | NA | NA | NA | 589 |
| **A*33:03~B*18:02~DRB1*12:02** | 0.0017 | 1 | 9.70 | 4.66 | 11.89 | NA | NA | NA | NA | NA |
| A*24:02~B*41:01~DRB1*04:02 | 0.0017 | 1 | 1.43 | 2.36 | 5.97 | NA | 1983 | NA | NA | NA |
| A*02:01~B*51:01~DRB1*08:04 | 0.0017 | 1 | 2.38 | 0.37 | 0.56 | NA | NA | NA | NA | NA |
| A*03:01~B*38:01~DRB1*14:01 | 0.0017 | 1 | 0.06 | 1.68 | 8.59 | NA | NA | NA | NA | NA |
| A*11:01~B*58:01~DRB1*04:05 | 0.0017 | 1 | 0.13 | 1.98 | 1.92 | NA | NA | NA | NA | NA |
| A*69:01~B*15:01~DRB1*13:02 | 0.0017 | 1 | 4.59 | 4.81 | 0.00 | NA | NA | NA | NA | NA |
| A*01:04N~B*49:01~DRB1*11:01 | 0.0017 | 1 | 5.65 | 2.96 | 0.32 | NA | NA | NA | NA | NA |
| A*29:02~B*14:02~DRB1*15:01 | 0.0017 | 1 | 1.22 | -0.68 | -0.09 | NA | 1163 | NA | NA | NA |
| A*33:01~B*40:02~DRB1*13:01 | 0.0017 | 1 | 3.15 | 1.19 | 2.13 | NA | NA | 1093 | NA | NA |
| A*24:02~B*58:01~DRB1*03:01 | 0.0017 | 1 | -0.12 | 0.63 | 2.16 | NA | NA | NA | 58 | NA |
| A*02:01~B*40:02~DRB1*07:01 | 0.0017 | 1 | -0.01 | -1.10 | -0.30 | NA | 316 | NA | NA | NA |
| A*32:01~B*51:01~DRB1*11:01 | 0.0017 | 1 | 0.36 | 1.66 | 3.47 | NA | NA | NA | NA | 359 |
| A*32:01~B*52:01~DRB1*07:01 | 0.0017 | 1 | 1.06 | 1.81 | 0.10 | NA | NA | NA | NA | NA |
| A*68:02~B*27:02~DRB1*16:01 | 0.0017 | 1 | 5.00 | 1.74 | 6.36 | NA | NA | NA | NA | NA |
| A*30:01~B*53:01~DRB1*13:01 | 0.0017 | 1 | 2.94 | 0.33 | 0.75 | NA | NA | NA | NA | NA |
| **A*33:03~B*53:01~DRB1*08:04** | 0.0017 | 1 | 3.73 | 3.73 | 3.73 | NA | 991 | 5 | NA | 252 |
| A*74:01~B*57:03~DRB1*11:01 | 0.0017 | 1 | 17.00 | 2.96 | 1.88 | NA | NA | 849 | NA | NA |
| A*03:01~B*08:01~DRB1*04:03 | 0.0017 | 1 | -0.92 | 2.98 | 1.56 | NA | NA | NA | NA | NA |
| A*11:01~B*49:01~DRB1*04:05 | 0.0017 | 1 | -0.14 | 1.98 | 1.57 | NA | 574 | NA | NA | 83 |
| A*29:02~B*27:05~DRB1*04:01 | 0.0017 | 1 | 0.84 | 1.91 | 0.84 | NA | NA | NA | NA | NA |
| A*24:02~B*44:02~DRB1*15:01 | 0.0017 | 1 | -0.99 | 0.67 | 0.43 | NA | 2472 | NA | NA | NA |
| A*02:01~B*35:03~DRB1*13:03 | 0.0017 | 1 | 0.46 | 0.39 | 1.06 | NA | NA | NA | NA | NA |
| A*24:03~B*15:01~DRB1*08:04 | 0.0017 | 1 | 4.59 | 9.70 | 1.44 | NA | NA | NA | NA | NA |
| A*26:01~B*58:01~DRB1*03:01 | 0.0017 | 1 | 0.36 | -0.77 | 2.16 | NA | NA | NA | NA | NA |
| **A*02:06~B*54:01~DRB1*04:05** | 0.0017 | 1 | 17.00 | 5.84 | 8.38 | NA | NA | NA | 95 | NA |
| **A*24:07~B*13:01~DRB1*12:02** | 0.0017 | 1 | 24.08 | 11.89 | 11.89 | NA | NA | NA | NA | NA |
| A*31:01~B*49:01~DRB1*11:01 | 0.0017 | 1 | 0.70 | -0.47 | 0.32 | NA | NA | NA | NA | NA |
| A*02:01~B*15:10~DRB1*03:01 | 0.0017 | 1 | 0.25 | 0.17 | 2.85 | NA | NA | 923 | NA | NA |
| **A*36:01~B*53:01~DRB1*03:02** | 0.0017 | 1 | 13.72 | 17.00 | 9.70 | NA | NA | 301 | NA | NA |
| A*02:01~B*57:01~DRB1*08:04 | 0.0017 | 1 | -0.91 | 0.37 | 1.72 | NA | NA | NA | NA | NA |
| **A*02:05~B*47:01~DRB1*13:03** | 0.0017 | 1 | 9.65 | 3.55 | 4.50 | NA | NA | NA | NA | NA |
| A*11:01~B*07:02~DRB1*15:02 | 0.0017 | 1 | -1.06 | 1.77 | 0.03 | NA | NA | NA | 599 | NA |
| A*02:01~B*49:01~DRB1*03:01 | 0.0017 | 1 | -0.15 | 0.17 | 0.08 | NA | NA | 520 | NA | NA |
| A*11:01~B*18:01~DRB1*16:02 | 0.0017 | 1 | 1.21 | 0.76 | 2.51 | NA | NA | NA | NA | 780 |
| A*03:01~B*18:01~DRB1*16:02 | 0.0017 | 1 | 0.39 | 0.11 | 2.51 | NA | NA | NA | NA | NA |
| A*23:01~B*73:01~DRB1*04:05 | 0.0017 | 1 | 6.51 | 1.92 | 8.38 | NA | NA | NA | NA | 901 |
| A*02:01~B*35:08~DRB1*04:07 | 0.0017 | 1 | 0.37 | 0.94 | 3.42 | NA | NA | NA | NA | NA |
| A*11:01~B*18:01~DRB1*13:01 | 0.0017 | 1 | 1.21 | 0.53 | -0.39 | NA | 1290 | NA | NA | NA |
| **A*36:01~B*53:01~DRB1*04:04** | 0.0017 | 1 | 13.72 | 4.31 | 2.22 | NA | NA | NA | NA | NA |
| **A*68:02~B*58:01~DRB1*10:01** | 0.0017 | 1 | 2.11 | 2.68 | 6.30 | NA | NA | 1814 | NA | 534 |
| A*03:01~B*18:05~DRB1*16:01 | 0.0017 | 1 | 2.44 | 1.26 | 5.65 | NA | NA | NA | NA | NA |
| LD: pairwise linkage disequilibrium as defined by standardized residuals; values >= 2 correspond to a significant association (shown in green); non-significant associations are shown in red. Haplotypes in complete linkage (i.e. across the three loci) are shown in bold. | | | | | | | | | | |
| Rank of haplotypes estimated on 6,114 volunteer donors from the Swiss registry (SBSC) and on four large groups (EUR: donors of European descent, AFA: donors of African descent, API: donors of Asian descent, HIS: donors of South American descent) of volunteer donors from the National Marrow Donor Program (NMDP) (Maiers et al. 2007). NA: not available. NA: not available. | | | | | | | | | | |

**Table S3** HLA-A~B~DRB1 phased haplotypes and linkage disequilibrium among allele pairs in the cohort of 291 patients
