# Supplementary material for: High-resolution HLA phased haplotype frequencies to predict the success of unrelated donor searches and clinical outcome following hematopoietic stem cell transplantation
Source: Bone Marrow Transplant. 2019 Apr 5;54(10):1701–9. doi: 10.1038/s41409-019-0520-6 (PMC7198472; doi:10.1038/s41409-019-0520-6)
Supplement: Supplementary file 4 — Table S4 [file 41409_2019_520_MOESM4_ESM.docx]

| **ABDR phased (n=291 patients)** | **freq** | **ABCDRDQ estimated by EM algorithm (n=6114 SBSC donors)** | **freq** | **LD BC^#^** | **LD DRDQ^#^** |
| --- | --- | --- | --- | --- | --- |
| A*01:01~B*08:01~DRB1*03:01 | 0.0326 | A*01:01:01G~B*08:01:01G~C*07:01:01G~DRB1*03:01:01G~DQB1*02:01:01G | 0.04148 | 67.69 | 60.18 |
|  |  | A*01:01:01G~B*08:01:02~C*07:01:01G~DRB1*03:01:01G~DQB1*02:01:01G | 0.00018 | 4.37 | 60.18 |
|  |  | A*01:01:01G~B*08:01:01G~C*07:07~DRB1*03:01:01G~DQB1*02:01:01G | 0.00016 | 5.10 | 60.18 |
|  |  | A*01:01:01G~B*08:01:01G~C*05:01:01G~DRB1*03:01:01G~DQB1*02:01:01G | 0.00008 | -8.22 | 60.18 |
|  |  | A*01:01:01G~B*08:01:01G~C*07:02:01G~DRB1*03:01:01G~DQB1*02:01:01G | 0.00005 | -8.50 | 60.18 |
| A*03:01~B*07:02~DRB1*15:01 | 0.0206 | A*03:01:01G~B*07:02:01G~C*07:02:01G~DRB1*15:01:01G~DQB1*06:02:01G | 0.02666 | 87.16 | 91.83 |
|  |  | A*03:01:01G~B*07:02:04~C*07:02:01G~DRB1*15:01:01G~DQB1*06:02:01G | 0.00090 | 8.16 | 91.83 |
|  |  | A*03:01:01G~B*07:02:01G~C*07:02:01G~DRB1*15:01:01G~DQB1*06:03:01G | 0.00050 | 87.16 | -7.70 |
|  |  | A*03:01:01G~B*07:02:01G~C*07:02:01G~DRB1*15:01:01G~DQB1*05:02:01G | 0.00018 | 87.16 | 0.58 |
|  |  | A*03:01:01G~B*07:02:01G~C*07:02:01G~DRB1*15:01:01G~DQB1*06:04:01G | 0.00008 | 87.16 | -8.12 |
|  |  | A*03:01:01G~B*07:02:01G~C*12:03:03~DRB1*15:01:01G~DQB1*05:02:01G | 0.00008 | 2.92 | 0.58 |
| A*02:01~B*07:02~DRB1*15:01 | 0.0172 | A*02:01:01G~B*07:02:01G~C*07:02:01G~DRB1*15:01:01G~DQB1*06:02:01G | 0.01167 | 87.16 | 91.83 |
|  |  | A*02:01:01G~B*07:02:01G~C*07:02:01G~DRB1*15:01:01G~DQB1*05:02:01G | 0.00072 | 87.16 | 0.58 |
|  |  | A*02:01:01G~B*07:02:01G~C*07:02:01G~DRB1*15:01:01G~DQB1*06:03:01G | 0.00017 | 87.16 | -7.70 |
|  |  | A*02:01:01G~B*07:02:01G~C*03:04:01G~DRB1*15:01:01G~DQB1*06:02:01G | 0.00012 | -8.00 | 91.83 |
|  |  | A*02:01:01G~B*07:02:01G~C*12:03:01G~DRB1*15:01:01G~DQB1*05:02:01G | 0.00009 | -7.49 | 0.58 |
|  |  | A*02:01:01G~B*07:02:01G~C*04:01:01G~DRB1*15:01:01G~DQB1*06:02:01G | 0.00008 | -13.00 | 91.83 |
|  |  | A*02:01:01G~B*07:02:01G~C*02:02:02G~DRB1*15:01:01G~DQB1*06:02:01G | 0.00006 | -7.55 | 91.83 |
| A*03:01~B*35:01~DRB1*01:01 | 0.0137 | A*03:01:01G~B*35:01:01G~C*04:01:01G~DRB1*01:01:01G~DQB1*05:01:01G | 0.01373 | 64.84 | 84.79 |
| A*01:01~B*57:01~DRB1*07:01 | 0.012 | A*01:01:01G~B*57:01:01G~C*06:02:01G~DRB1*07:01:01G~DQB1*03:03:02G | 0.00779 | 49.48 | 40.96 |
|  |  | A*01:01:01G~B*57:01:01G~C*07:01:01G~DRB1*07:01:01G~DQB1*03:03:02G | 0.00038 | -2.26 | 40.96 |
|  |  | A*01:01:01G~B*57:01:01G~C*06:02:01G~DRB1*07:01:01G~DQB1*02:01:01G | 0.00006 | 49.48 | 51.12 |
| A*29:02~B*44:03~DRB1*07:01 | 0.012 | A*29:02:01G~B*44:03:01G~C*16:01:01G~DRB1*07:01:01G~DQB1*02:01:01G | 0.01201 | 71.65 | 51.12 |
|  |  | A*29:02:01G~B*44:03:01G~C*16:01:01G~DRB1*07:01:01G~DQB1*03:03:02G | 0.00033 | 71.65 | 40.96 |
|  |  | A*29:02:01G~B*44:03:01G~C*04:01:01G~DRB1*07:01:01G~DQB1*02:01:01G | 0.00016 | 17.46 | 51.12 |
| A*26:01~B*38:01~DRB1*13:01 | 0.0103 | A*26:01:01G~B*38:01:01~C*12:03:01G~DRB1*13:01:01G~DQB1*06:03:01G | 0.00284 | 64.21 | 99.16 |
| A*01:01~B*08:01~DRB1*15:01 | 0.0086 | A*01:01:01G~B*08:01:01G~C*07:01:01G~DRB1*15:01:01G~DQB1*06:02:01G | 0.00459 | 67.69 | 91.83 |
|  |  | A*01:01:01G~B*08:01:01G~C*07:02:01G~DRB1*15:01:01G~DQB1*06:02:01G | 0.00008 | -8.50 | 91.83 |
|  |  | A*01:01:01G~B*08:01:02~C*07:01:01G~DRB1*15:01:01G~DQB1*06:02:01G | 0.00003 | 4.37 | 91.83 |
| A*02:01~B*44:02~DRB1*04:01 | 0.0086 | A*02:01:01G~B*44:02:01G~C*05:01:01G~DRB1*04:01~DQB1*03:01:01G | 0.01252 | 76.31 | 14.64 |
|  |  | A*02:01:01G~B*44:02:01G~C*05:01:01G~DRB1*04:01~DQB1*03:02:01G | 0.00229 | 76.31 | 42.50 |
|  |  | A*02:01:01G~B*44:02:01G~C*12:03:01G~DRB1*04:01~DQB1*03:01:01G | 0.00016 | -6.90 | 14.64 |
| A*02:01~B*51:01~DRB1*11:01 | 0.0086 | A*02:01:01G~B*51:01:01G~C*15:02:01G~DRB1*11:01:01G~DQB1*03:01:01G | 0.00221 | 56.44 | 52.67 |
|  |  | A*02:01:01G~B*51:01:01G~C*14:02:01G~DRB1*11:01:01G~DQB1*03:01:01G | 0.00163 | 43.70 | 52.67 |
|  |  | A*02:01:01G~B*51:01:01G~C*02:02:02G~DRB1*11:01:01G~DQB1*03:01:01G | 0.00099 | 3.51 | 52.67 |
|  |  | A*02:01:01G~B*51:01:01G~C*16:02:01G~DRB1*11:01:01G~DQB1*03:01:01G | 0.00062 | 13.92 | 52.67 |
|  |  | A*02:01:01G~B*51:01:01G~C*01:02:01G~DRB1*11:01:01G~DQB1*03:01:01G | 0.00038 | 14.14 | 52.67 |
|  |  | A*02:01:01G~B*51:01:01G~C*05:01:01G~DRB1*11:01:01G~DQB1*03:01:01G | 0.00013 | -3.75 | 52.67 |
|  |  | A*02:01:01G~B*51:01:01G~C*15:06:01~DRB1*11:01:01G~DQB1*03:01:01G | 0.00008 | 9.27 | 52.67 |
|  |  | A*02:01:01G~B*51:01:01G~C*12:03:01G~DRB1*11:01:01G~DQB1*03:01:01G | 0.00008 | -4.63 | 52.67 |
|  |  | A*02:01:01G~B*51:01:01G~C*14:04~DRB1*11:01:01G~DQB1*03:01:01G | 0.00008 | 4.96 | 52.67 |
| A*24:02~B*07:02~DRB1*15:01 | 0.0086 | A*24:02:01G~B*07:02:01G~C*07:02:01G~DRB1*15:01:01G~DQB1*06:02:01G | 0.00632 | 87.16 | 91.83 |
|  |  | A*24:02:01G~B*07:02:01G~C*07:02:01G~DRB1*15:01:01G~DQB1*06:03:01G | 0.00018 | 87.16 | -7.70 |
|  |  | A*24:02:01G~B*07:02:01G~C*07:02:01G~DRB1*15:01:01G~DQB1*06:81 | 0.00008 | 87.16 | 2.83 |
|  |  | A*24:02:01G~B*07:02:01G~C*12:03:01G~DRB1*15:01:01G~DQB1*06:02:01G | 0.00008 | -7.49 | 91.83 |
|  |  | A*24:02:01G~B*07:02:01G~C*07:02:01G~DRB1*15:01:01G~DQB1*05:03:01G | 0.00008 | 87.16 | -6.99 |
| A*24:02~B*08:01~DRB1*03:01 | 0.0086 | A*24:02:01G~B*08:01:01G~C*07:01:01G~DRB1*03:01:01G~DQB1*02:01:01G | 0.00224 | 67.69 | 60.18 |
|  |  | A*24:02:01G~B*08:01:01G~C*07:02:01G~DRB1*03:01:01G~DQB1*02:01:01G | 0.00033 | -8.50 | 60.18 |
| A*30:01~B*13:02~DRB1*07:01 | 0.0086 | A*30:01:01G~B*13:02:01G~C*06:02:01G~DRB1*07:01:01G~DQB1*02:01:01G | 0.00809 | 56.12 | 51.12 |
| A*02:01~B*15:01~DRB1*04:01 | 0.0069 | A*02:01:01G~B*15:01:01G~C*03:04:01G~DRB1*04:01~DQB1*03:02:01G | 0.00610 | 22.27 | 42.50 |
|  |  | A*02:01:01G~B*15:01:01G~C*03:03:01G~DRB1*04:01~DQB1*03:02:01G | 0.00232 | 58.88 | 42.50 |
|  |  | A*02:01:01G~B*15:01:01G~C*01:02:01G~DRB1*04:01~DQB1*03:01:01G | 0.00021 | 5.14 | 14.64 |
| A*02:01~B*18:01~DRB1*03:01 | 0.0069 | A*02:01:01G~B*18:01:01G~C*05:01:01G~DRB1*03:01:01G~DQB1*02:01:01G | 0.00186 | 10.92 | 60.18 |
| A*01:01~B*15:17~DRB1*13:02 | 0.0052 | A*01:01:01G~B*15:17:01G~C*07:01:01G~DRB1*13:02:01~DQB1*06:04:01G | 0.00069 | 19.54 | 98.67 |
| A*02:01~B*08:01~DRB1*03:01 | 0.0052 | A*02:01:01G~B*08:01:01G~C*07:01:01G~DRB1*03:01:01G~DQB1*02:01:01G | 0.00550 | 67.69 | 60.18 |
|  |  | A*02:01:01G~B*08:01:01G~C*07:02:01G~DRB1*03:01:01G~DQB1*02:01:01G | 0.00036 | -8.50 | 60.18 |
| A*02:01~B*13:02~DRB1*13:01 | 0.0052 | A*02:01:01G~B*13:02:01G~C*06:02:01G~DRB1*13:01:01G~DQB1*06:03:01G | 0.00064 | 56.12 | 99.16 |
| A*02:01~B*18:01~DRB1*11:04 | 0.0052 | A*02:01:01G~B*18:01:01G~C*07:01:01G~DRB1*11:04~DQB1*03:01:01G | 0.00444 | 20.14 | 37.56 |
| A*02:01~B*40:01~DRB1*13:02 | 0.0052 | A*02:01:01G~B*40:01:01G~C*03:04:01G~DRB1*13:02:01~DQB1*06:04:01G | 0.00957 | 82.52 | 98.67 |
|  |  | A*02:01:01G~B*40:01:01G~C*07:02:01G~DRB1*13:02:01~DQB1*06:04:01G | 0.00009 | -6.94 | 98.67 |
|  |  | A*02:01:01G~B*40:01:01G~C*06:02:01G~DRB1*13:02:01~DQB1*06:04:01G | 0.00008 | -7.04 | 98.67 |
|  |  | A*02:01:01G~B*40:01:01G~C*03:04:01G~DRB1*13:02:01~DQB1*06:02:01G | 0.00008 | 82.52 | -8.33 |
| A*02:01~B*51:01~DRB1*08:01 | 0.0052 | A*02:01:01G~B*51:01:01G~C*14:02:01G~DRB1*08:01:01G~DQB1*04:02:01G | 0.00147 | 43.70 | 97.23 |
|  |  | A*02:01:01G~B*51:01:01G~C*15:02:01G~DRB1*08:01:01G~DQB1*04:02:01G | 0.00103 | 56.44 | 97.23 |
|  |  | A*02:01:01G~B*51:01:01G~C*04:01:01G~DRB1*08:01:01G~DQB1*04:02:01G | 0.00055 | -8.92 | 97.23 |
|  |  | A*02:01:01G~B*51:01:01G~C*02:02:02G~DRB1*08:01:01G~DQB1*04:02:01G | 0.00021 | 3.51 | 97.23 |
|  |  | A*02:01:01G~B*51:01:01G~C*05:01:01G~DRB1*08:01:01G~DQB1*04:02:01G | 0.00015 | -3.75 | 97.23 |
| A*31:01~B*40:01~DRB1*04:04 | 0.0052 | A*31:01:02G~B*40:01:01G~C*03:04:01G~DRB1*04:04:01~DQB1*03:02:01G | 0.00413 | 82.52 | 58.27 |
| A*32:01~B*44:03~DRB1*07:01 | 0.0052 | A*32:01:01G~B*44:03:01G~C*16:01:01G~DRB1*07:01:01G~DQB1*02:01:01G | 0.00074 | 71.65 | 51.12 |
|  |  | A*32:01:01G~B*44:03:01G~C*04:01:01G~DRB1*07:01:01G~DQB1*02:01:01G | 0.00051 | 17.46 | 51.12 |
| SBSC: Swiss Blood Stem Cells registry; EM: expectation-maximization; LD: linkage disequilibrium; freq: frequency; | | |  |  |  |
| ^#^: linkage disequilibrium between HLA-B and C, on the one hand, and between DRB1 and DQB1, on the other hand, assessed by standardized residuals, where a value of plus or minus 2 indicates a significant deviation under the assumption of random association. Positive values for coupled alleles and negative values for repulsive alleles. | | | | | |

**Table S4** Most frequent phased HLA-A~B~DRB1 haplotypes (observed at least three times) in the cohort of 291 patients and their corresponding five locus haplotypes and HLA-B~C and DRB1~DQB1 linkage disequilibrium patterns in Swiss donors (n=6,114)
