# Supplementary material for: High-resolution HLA phased haplotype frequencies to predict the success of unrelated donor searches and clinical outcome following hematopoietic stem cell transplantation
Source: Bone Marrow Transplant. 2019 Apr 5;54(10):1701–9. doi: 10.1038/s41409-019-0520-6 (PMC7198472; doi:10.1038/s41409-019-0520-6)
Supplement: Supplementary file 5 — Table S5 [file 41409_2019_520_MOESM5_ESM.docx]

|  |  |  |  |  |  |  | 95.0% CI for Exp(B) | |
| --- | --- | --- | --- | --- | --- | --- | --- | --- |
| Explanatory variable | B | SE | Wald | df | Sig. | Exp(B) | Lower | Upper |
| TX center |  |  | 23.731 | 3 | 0 |  |  |  |
| 2 | -0.706 | 0.349 | 4.095 | 1 | 0.043 | 0.494 | 0.249 | 0.978 |
| 3 | 0.989 | 0.311 | 10.116 | 1 | 0.001 | 2.689 | 1.462 | 4.947 |
| 4 | 0.755 | 0.687 | 1.208 | 1 | 0.272 | 2.127 | 0.554 | 8.173 |
| age |  |  | 11.721 | 3 | 0.008 |  |  |  |
| age (20-40) | 1.067 | 0.547 | 3.801 | 1 | 0.051 | 2.908 | 0.994 | 8.503 |
| age (40-60) | 1.036 | 0.471 | 4.842 | 1 | 0.028 | 2.818 | 1.12 | 7.092 |
| age (>60) | 1.761 | 0.535 | 10.856 | 1 | 0.001 | 5.821 | 2.041 | 16.598 |
| disease stage |  |  | 9.63 | 2 | 0.008 |  |  |  |
| disease stage (intermediate) | 0.256 | 0.283 | 0.814 | 1 | 0.367 | 1.291 | 0.741 | 2.251 |
| disease stage (advanced) | 0.974 | 0.32 | 9.26 | 1 | 0.002 | 2.649 | 1.414 | 4.962 |
| DPB1 MM |  |  | 0.615 | 2 | 0.735 |  |  |  |
| DPB1 MM (1 MM) | 0.051 | 0.322 | 0.025 | 1 | 0.874 | 1.052 | 0.56 | 1.976 |
| DPB1 MM (2 MM) | -0.172 | 0.351 | 0.24 | 1 | 0.624 | 0.842 | 0.423 | 1.675 |
| geno20 | -0.376 | 0.249 | 2.279 | 1 | 0.131 | 0.687 | 0.422 | 1.119 |
| Baseline for TX center = 1, for age = <20, for disease stage = early, for DPB1 MM = 0 MM | | | | | | | | |

**Table S5** Cox regression model for overall survival with geno20
