# Supplementary material for: High-resolution HLA phased haplotype frequencies to predict the success of unrelated donor searches and clinical outcome following hematopoietic stem cell transplantation
Source: Bone Marrow Transplant. 2019 Apr 5;54(10):1701–9. doi: 10.1038/s41409-019-0520-6 (PMC7198472; doi:10.1038/s41409-019-0520-6)
Supplement: Supplementary file 6 — Supplementary Figures legends [file 41409_2019_520_MOESM6_ESM.docx]

**Supplementary Figures**

**Figure S1**

Schematic representation of the data formatting and analyses. The HLA-A~B~DRB1 data of 843 patients and 2132 family members were first phased by segregation analysis. The patients’ haplotypes were then parsed with the help of GNU/Linux scripts to retain (1) unrelated individuals with haplotypes unambiguously defined by segregation analysis and (2) high resolution typing data (n=291 patients retained for the analyses). The estimated frequencies in a large cohort of 6114 unrelated volunteer donors from the Swiss registry (SBSC) were used for ranking haplotypes in the patients and predicting unrelated search outcome. The SBSC data were also used for the descriptive analyses on haplotypes and linkage disequilibrium patterns. In the last part of the study, clinical outcome in recipients transplanted with 10/10 MUD was investigated in a cohort of patients (n=211) from the four Swiss transplant centers for allogeneic HSCT. HLA haplotypes were also phased by segregation analysis in the additional recipients included in the clinical cohort thanks to family data.

**Figure S2**

Bar charts showing the most frequent HLA-A, B, DRB1 allele combinations observed in the cohort of 291 patients and whether these alleles are carried on the same chromosome (i.e. as a real haplotype) or co-occur only at the genotypic level.

**Figure S3**

ROC curve analysis with the sum of haplotype ranks used as a predictive variable for unrelated search outcome. The circled dot represents a cut-off value of 1000 for the sum and is a good trade-off between true and false positive rates.

**Figure S4**

Cumulative incidence for haplotypes groups according to relapse, chronic and acute GVHD. Regarding acute GVHD, the date of onset was only introduced in the database from 2013/2014 onwards and as such the interval to develop the disease is not calculated for several patients. Instead, a median value of 24 days for developing grade 2 to 4 GVHD was considered in the analysis based on available information.
