# Supplementary material for: High-resolution HLA phased haplotype frequencies to predict the success of unrelated donor searches and clinical outcome following hematopoietic stem cell transplantation
Source: Bone Marrow Transplant. 2019 Apr 5;54(10):1701–9. doi: 10.1038/s41409-019-0520-6 (PMC7198472; doi:10.1038/s41409-019-0520-6)
Supplement: Supplementary file 7 — Figure S1 [file 41409_2019_520_MOESM7_ESM.pdf]

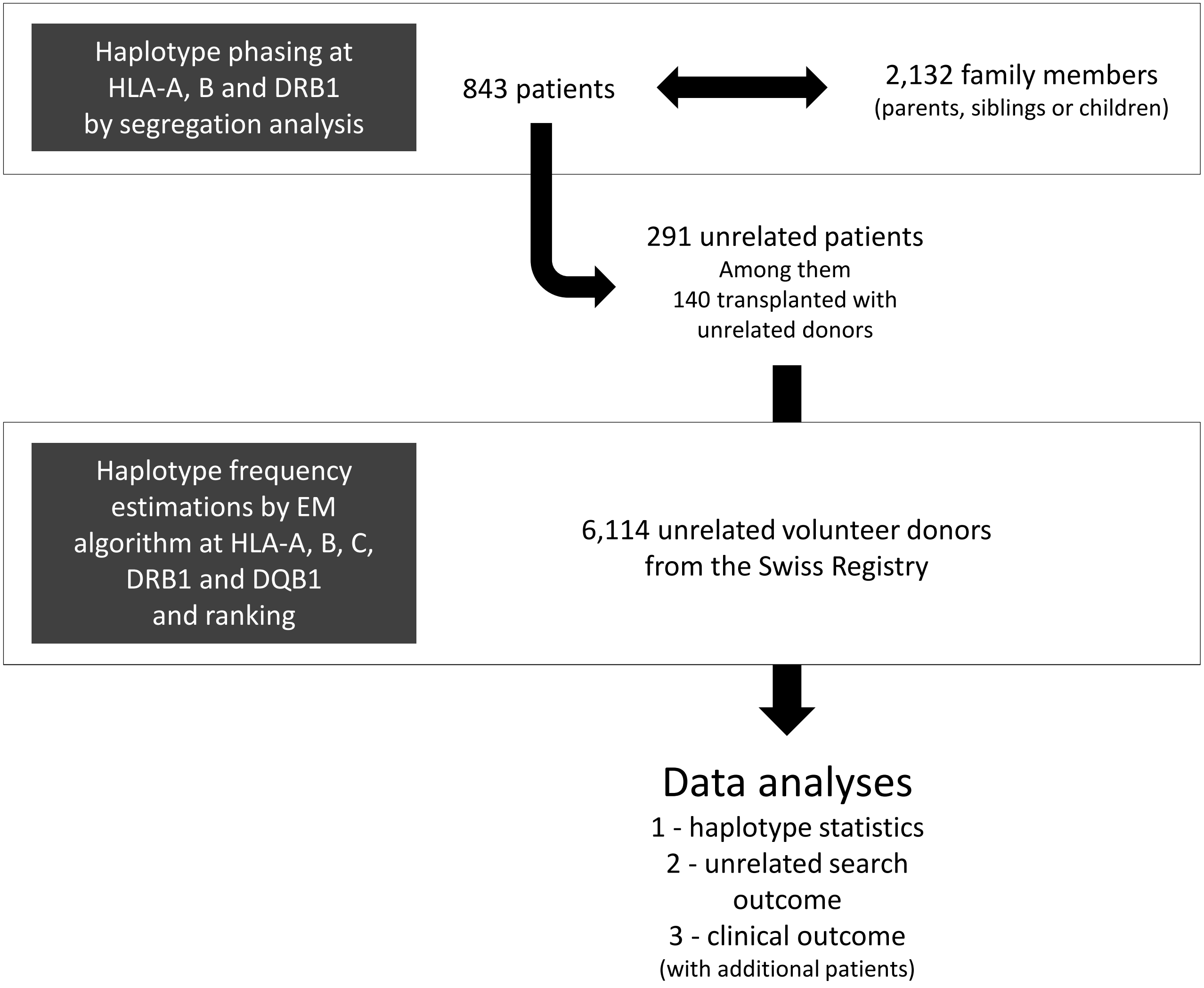
Haplotype phasing at HLA-A, B and DRB1 by segregation analysis
843 patients
2,132 family members
(parents, siblings or children)
291 unrelated patients
Among them 140 transplanted with unrelated donors
Haplotype frequency estimations by EM algorithm at HLA-A, B, C, DRB1 and DQB1 and ranking
6,114 unrelated volunteer donors from the Swiss Registry
Data analyses
1 - haplotype statistics
2 - unrelated search outcome
3 - clinical outcome
(with additional patients)
