# Supplementary figures and images for: High-resolution HLA phased haplotype frequencies to predict the success of unrelated donor searches and clinical outcome following hematopoietic stem cell transplantation
Source: Bone Marrow Transplant. 2019 Apr 5;54(10):1701–9. doi: 10.1038/s41409-019-0520-6 (PMC7198472; doi:10.1038/s41409-019-0520-6)

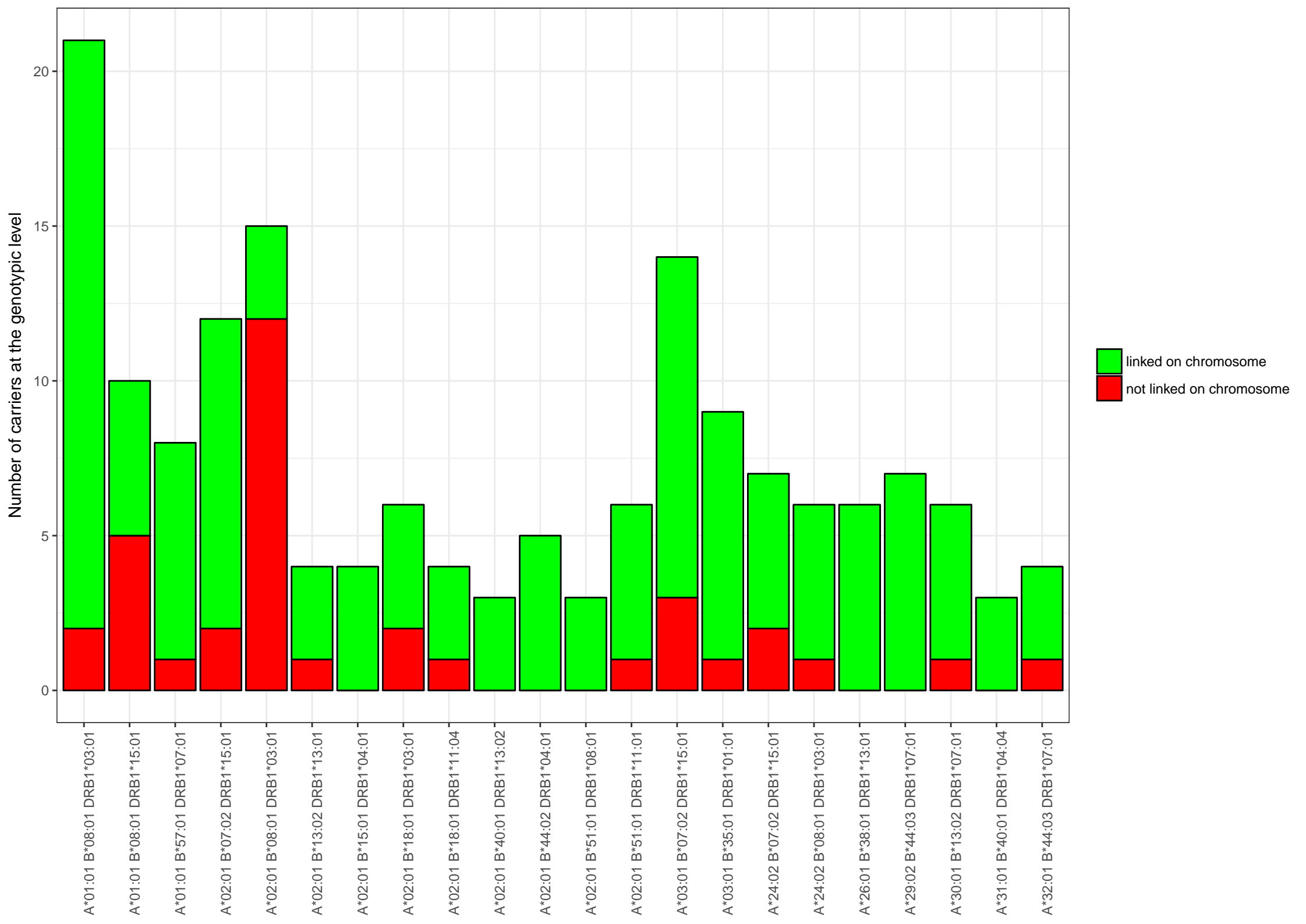

Supplement: Supplementary file 8 — Figure S2 [file 41409_2019_520_MOESM8_ESM.pdf]

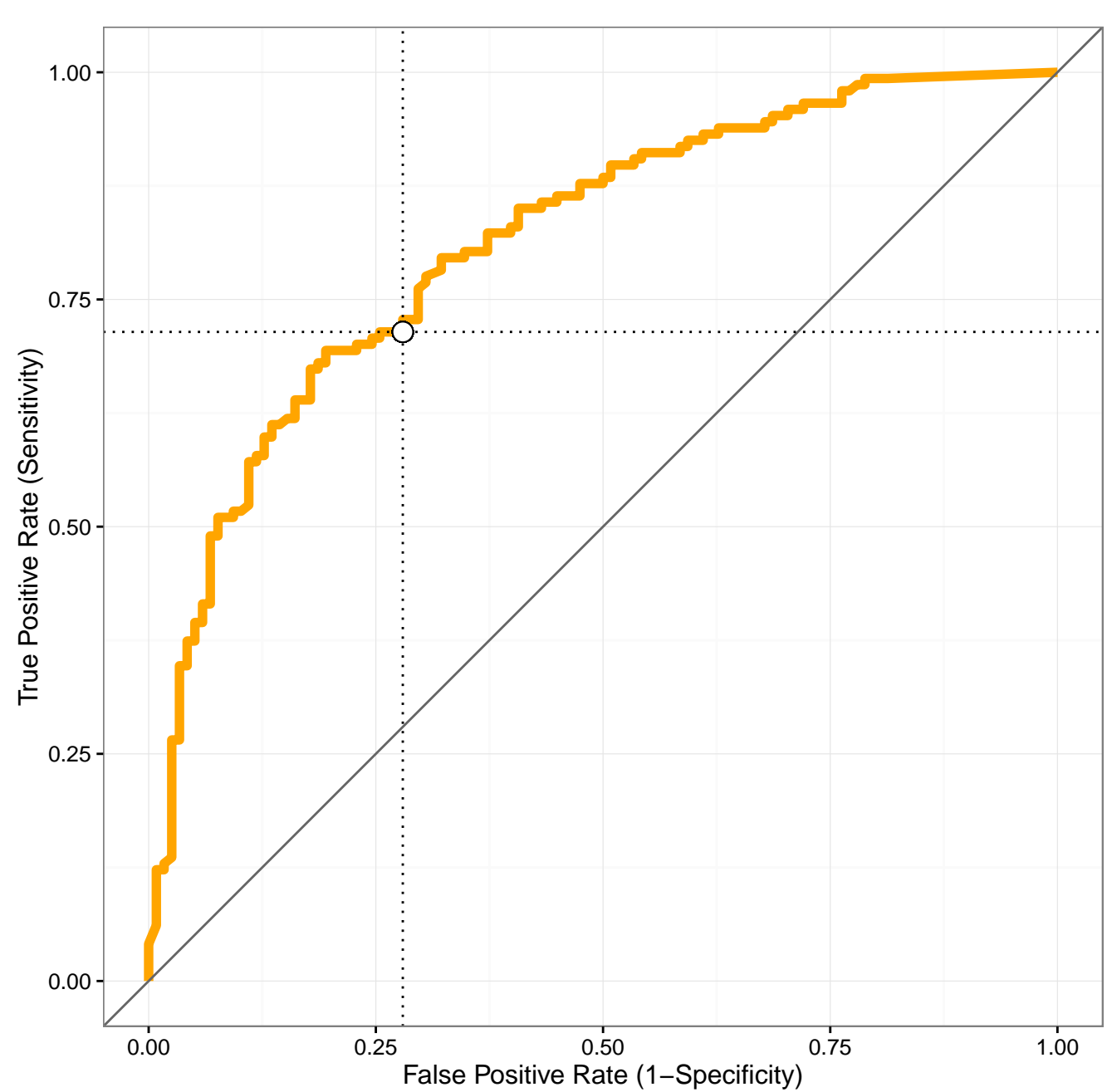

Supplement: Supplementary file 9 — Figure S3 [file 41409_2019_520_MOESM9_ESM.pdf]

## Relapse

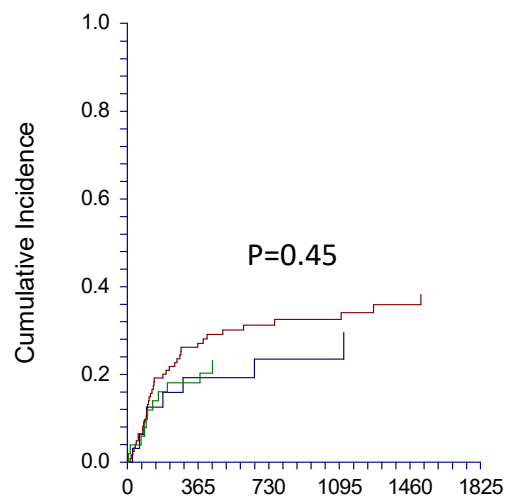

## cGVHD

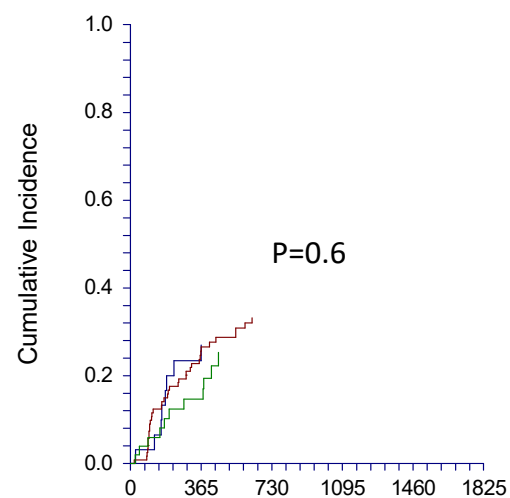

## aGVHD

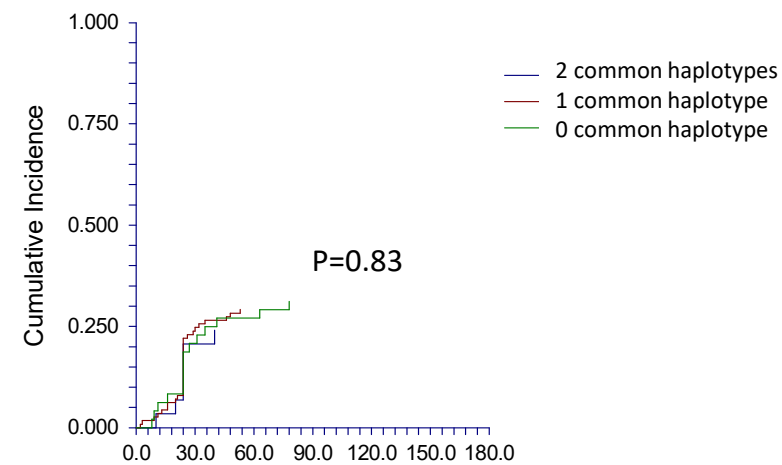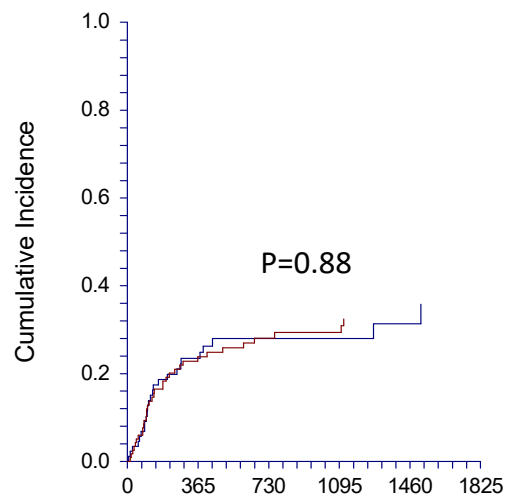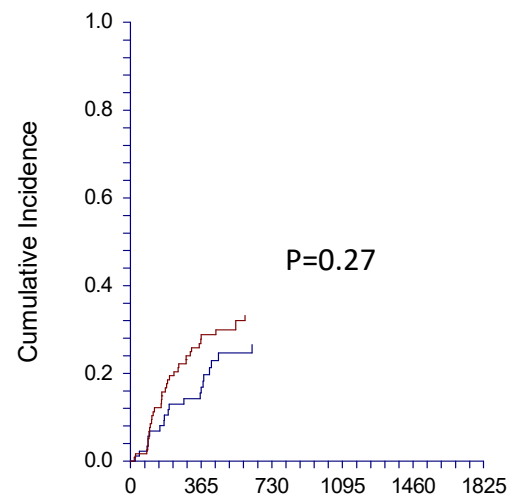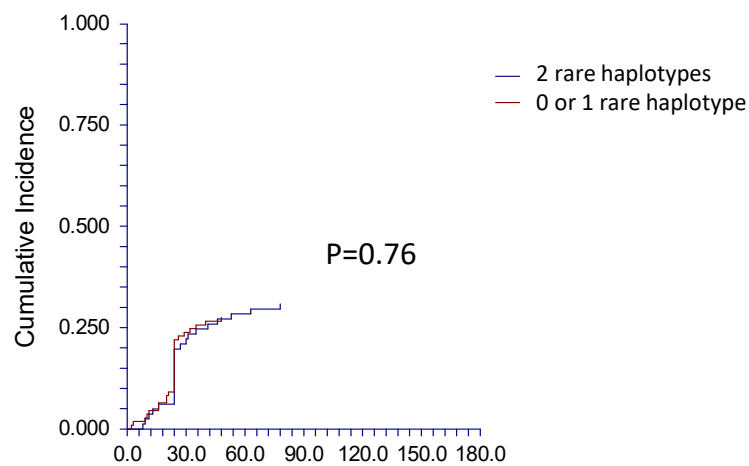

Supplement: Supplementary file 10 — Figure S4 [file 41409_2019_520_MOESM10_ESM.pdf]
